# Least squares and maximum likelihood estimation of sufficient reductions in regressions with matrix-valued predictors

**DOI:** 10.1007/s41060-020-00228-y

**Published:** 2020-08-04

**Authors:** Ruth M. Pfeiffer, Daniel B. Kapla, Efstathia Bura

**Affiliations:** 1 Biostatistics Branch, DCEG, National Cancer Institute, NIH, Bethesda, USA; 2 Faculty of Mathematics, Institute of Statistics and Mathematical Methods in Economics, TU Wien, Vienna, Austria

**Keywords:** Dimension, Reduction, Regression, Classification

## Abstract

We propose methods to estimate sufficient reductions in matrix-valued predictors for regression or classification. We assume that the first moment of the predictor matrix given the response can be decomposed into a *row* and *column* component via a Kronecker product structure. We obtain least squares and maximum likelihood estimates of the sufficient reductions in the matrix predictors, derive statistical properties of the resulting estimates and present fast computational algorithms with assured convergence. The performance of the proposed approaches in regression and classification is compared in simulations.We illustrate the methods on two examples, using longitudinally measured serum biomarker and neuroimaging data.

## Introduction

In many applications, predictors are matrix-valued. For example, in cohort studies conducted to study diseases, multiple correlated biomarkers are measured repeatedly during follow-up. It is of interest to assess their associations with disease outcomes to aid understanding of biological underpinnings of disease and to use them individually or in combinations in diagnostic or prognostic models. Neuroimaging studies use data from electroencephalography (EEG) that records electrical activity of the brain over time, to predict cognitive outcomes and to identify brain regions associated with a clinical response. In these examples, the predictor vectors measured at different time points can be represented as a matrix.

Multivariate statistical methods can be used to analyze matrix-valued predictors by mapping them into vectors. Frequently this is not feasible for data sets of realistic size. For instance, treating EEG data measured at 60 channels each for 256 time points as a vector in a regression model would require estimating 15360 regression parameters, necessitating practically impossibly large samples. Moreover, vectorizing a matrix destroys the inherent structure of the predictors that may contain important modeling information.

Only few statistical approaches accommodate a matrix structure of the predictors. *Dimension folding* [29] extends moment-based sufficient dimension reduction (SDR) methods for matrix-valued predictors by reducing the predictors’ row and column dimensions simultaneously without loss of information on the response. [34] proposed and studied first-moment-based SDR methods for combining several longitudinally measured predictors into a composite score for prediction or regression modeling. They assumed that the means and the second moments of the predictors can be separated into a predictor-specific and a time-specific component via a Kronecker product structure and proposed an estimation approach, longitudinal sliced inverse regression (LSIR), based on empirical moments of the predictors given the outcome. The Kronecker product structure substantially reduces the complexity of the first-moment-based dimension reduction subspace. The resulting score yielded better predictive accuracy than standard first-moment-based SDR methods, such as sliced inverse regression (SIR) [31], applied to the vectorized predictors.

[17] developed model-based methods, *dimension folding principal component analysis (PCA)* and *dimension folding principal fitted components (PFC)*, that extend conventional PCA and PFC [12] to matrix-valued data. They require the predictors be normally distributed with Kronecker product covariance structure. In the context of classification, [32] proposed a discriminant analysis model to predict a categorical response for mixed categorical and tensor-valued predictors. The method reduces the dimension of the tensor predictor within each group defined by the categorical covariates.

In the machine learning literature, methods proposed for matrix-valued predictors that do not use information on outcome; i.e., unsupervised dimension reduction methods include 2DPCA [41], generalized 2D principal component analysis (G2DPCA) [27], (2D)$^2$PCA [45], GLRAM [42], unified PCA [37] and probabilistic higher-order PCA [44]. Regression approaches include reduced-rank generalized linear models using a mixture of array-valued and vector-valued predictors [47] and a tensor partial least squares algorithm for the regression of a continuous response on tensor-valued predictors [46]. Both focus on the forward regression which they assume is linear in the vector-, matrix- or tensor-valued predictors. These methods frequently suffer from lack of convergence and do not yield closed form solutions.

In this paper, we propose least squares and maximum likelihood-based approaches to estimate the sufficient reductions in matrix-valued predictors under a Kronecker product structure for the predictor means given the response without requiring a specific structure for the covariance in contrast to previous methods [17, 34]. By casting the estimation problem in a linear model framework, we obtain least squares-based estimates that are asymptotically optimal and competitive with maximum likelihood estimates (MLEs) for practically relevant sample sizes.

## Background on sufficient dimension reduction

Let $\mathbf {X}=(X_1,\ldots ,X_p)^T \in {{\mathbb {R}}}^p$ be a vector of *p* predictors and $Y \in {{\mathbb {R}}}$ denote the outcome variable. sufficient dimension reduction, SDR [9], aims to find a function or “reduction” of $\mathbf {X}$, $\mathbf {R}: {{\mathbb {R}}}^{p} \rightarrow {{\mathbb {R}}}^d$ with $d \le p$, which contains the same information as $\mathbf {X}$ about the response *Y*. That is, $F(Y\mid \mathbf {X})=F(Y\mid \mathbf {R}(\mathbf {X}))$, where *F* is the conditional distribution function of *Y* given $\mathbf {X}$. This version of dimension reduction is called *sufficient* because the lower-dimensional $\mathbf {R}(\mathbf {X})$ ($d < p$) replaces the predictor vector $\mathbf {X}$ without any loss of information on *Y*. The dimension *d* of the sufficient reduction $\mathbf {R}(\mathbf {X})$ is the dimension of the regression of *Y* on $\mathbf {X}$.

With few exceptions [5, 21, 28], mostly *linear* sufficient reductions, $\mathbf {R}(\mathbf {X}) = \varvec{\eta }^T \mathbf {X}$, $\varvec{\eta }\in {{\mathbb {R}}}^{p \times d}$, have been studied in the SDR literature [e.g., [3, 8–10, 14, 31]]. Linear reductions are not unique.^1^ Therefore, in *linear SDR* the target is the subspace ${\mathcal {S}}(\varvec{\eta })={\text {span}}(\varvec{\eta })$, where $\varvec{\eta }$ is any basis of ${\mathcal {S}}(\varvec{\eta })$ satisfying $F(Y\mid \mathbf {X})=F(Y\mid \varvec{\eta }^T\mathbf {X})$.

Early SDR methods estimated sufficient reductions using kernel or *core* matrices $\varvec{\Omega }$ with ${\text {span}}(\varvec{\Omega }) \subseteq {\mathcal {S}}(\varvec{\eta })$. Because $\varvec{\Omega }$ is computed from moments of the conditional distribution of $\mathbf {X}\mid Y$, this version of SDR is called *moment-based* SDR (see, e.g., [3, 7, 14, 30, 31]).

*Model-based* SDR is based on the important result that if $\mathbf {R}(\mathbf {X})$ is a sufficient reduction for the forward regression $Y \mid \mathbf {X}$, then it is also a sufficient statistic for the inverse regression $\mathbf {X}\mid Y$ [11]. Exploiting this, both linear and nonlinear sufficient reductions for the regression of *Y* on $\mathbf {X}$ have been derived by requiring the distribution of $\mathbf {X}\mid Y$ be in the elliptically contoured or exponential family [4, 5, 11–13].

### First-moment-based SDR subspace

In this paper, we focus on inference on the first-moment-based $\text {SDR}$ subspace ($\text {FMSDR}$), which is the span of the centered mean of the inverse regression of $\mathbf {X}$ on *Y*, ${\mathbb {E}}(\mathbf {X}\mid Y) - {\mathbb {E}}(\mathbf {X})$, scaled by the inverse of the marginal covariance of $\mathbf {X}$, $\varvec{\varSigma }_{\mathbf {x}}$. That is, we let

1 $$\begin{aligned} {\mathcal {S}}_{\text {FMSDR}}&= \varvec{\varSigma }_{\mathbf {x}}^{-1} {\text {span}}\left( \varvec{\mu }_Y - \varvec{\mu }\right) , \end{aligned}$$

where $\varvec{\mu }_Y = {\mathbb {E}}(\mathbf {X}\mid Y)$ and $\varvec{\mu }= {\mathbb {E}}(\mathbf {X})$. If the predictors $\mathbf {X}$ satisfy the *linearity condition* [9, p.188] that requires ${\mathbb {E}}(\mathbf {X}\mid \varvec{\eta }^T\mathbf {X})$ be linear in $\varvec{\eta }^T\mathbf {X}$ for $\varvec{\eta }$ such that $F(Y\mid \mathbf {X})=F(Y\mid \varvec{\eta }^T \mathbf {X})$, then ${\mathcal {S}}_{\text {FMSDR}} \subseteq {\mathcal {S}}(\varvec{\eta }).$ The linearity condition refers exclusively to the marginal distribution of $\mathbf {X}$. It holds when $\mathbf {X}$ has an elliptical distribution, such as multivariate normal or multivariate *t*, and also holds approximately when *p* is very large [24, 38].

Under the linearity condition, any *core* matrix $\varvec{\Omega }$ whose column space spans the same space as ${\mathcal {S}}_{\text {FMSDR}}$ can be used to either exhaustively or partially estimate ${\mathcal {S}}(\varvec{\eta })$. SDR methods based on the first conditional moment of the inverse predictors $\mathbf {X}\mid Y$, such as SIR [31], use $\varvec{\Omega }=\varvec{\varSigma }_{\mathbf {x}}^{-1}{\mathbb {V}}\text {ar}({\mathbb {E}}(\mathbf {X}\mid Y))$.

[3] proposed *parametric inverse regression* (PIR) to obtain a least squares estimate of ${\mathcal {S}}(\varvec{\eta })$ from fitting the multivariate linear inverse regression model

2 $$\begin{aligned} \mathbf {X}&= \varvec{\mu }+ \mathbf {B}\mathbf {f}_Y +\varvec{\varepsilon }, \end{aligned}$$

where $\mathbf {f}_Y$ is an $r \times 1$ vector of functions of *Y* with ${\mathbb {E}}( \mathbf {f}_Y) =0$, the $p \times r$ unknown parameter matrix $\mathbf {B}$ is unconstrained, ${\mathbb {E}}(\varvec{\varepsilon }\mid Y)=0$ and ${\mathbb {V}}\text {ar}(\varvec{\varepsilon }\mid Y)={\mathbb {V}}\text {ar}(\mathbf {X}\mid Y)=\varvec{\varDelta }_Y$.

Model (2) implies ${\mathbb {E}}(\mathbf {X}\mid Y=y)=\varvec{\mu }_y= \varvec{\mu }+ \mathbf {B}\mathbf {f}_y$, and thus, ${\mathcal {S}}_{\text {FMSDR}}=\varvec{\varSigma }_{\mathbf {x}}^{-1}{\text {span}}(\mathbf {B})$, which is estimated from a random sample $(Y_i,\mathbf {X}_i^T), i=1,\ldots , n$, as follows. Let ${\mathbb {X}}$ denote the $n \times p$ matrix with rows $(\mathbf {X}_i - {\overline{\mathbf {X}}})^T$, where ${\overline{\mathbf {X}}}=\sum _{i=1}^n \mathbf {X}_i/n$, and ${\mathbb {F}}$ is the $n \times r$ matrix with rows $(\mathbf {f}_{y_i} -{\bar{\mathbf {f}}})^T$, with ${\bar{\mathbf {f}}}=\sum _{i=1}^n \mathbf {f}_{y_i}/n$. Regressing ${\mathbb {X}}$ on ${\mathbb {F}}$ yields the ordinary least squares (OLS) estimate for $\mathbf {B}$,

3 $$\begin{aligned} {\widehat{\mathbf {B}}}&=({\mathbb {F}}^T {\mathbb {F}})^{-1}{\mathbb {F}}^T{\mathbb {X}} \end{aligned}$$

in model (2). Letting $\mathbf {P}_{{\mathbb {F}}}={\mathbb {F}}({\mathbb {F}}^T {\mathbb {F}})^{-1}{\mathbb {F}}^T$ denote the projection matrix onto the space spanned by the columns of ${\mathbb {F}}$, an estimate of the matrix $\varvec{\varDelta }_Y$ is

4 $$\begin{aligned} {\widehat{{\mathbb {V}}\text {ar}}}(\mathbf {X}\mid Y)&= \frac{{\mathbb {X}}^T(\mathbf {I}-\mathbf {P}_{{\mathbb {F}}}){\mathbb {X}}}{n-{\text {rank}}({\mathbb {F}})}=\frac{{\mathbb {X}}^T\mathbf {Q}_{{\mathbb {F}}}{\mathbb {X}}}{n-{\text {rank}}({\mathbb {F}})}, \end{aligned}$$

where $\mathbf {Q}_{{\mathbb {F}}} = \mathbf {I}_n - \mathbf {P}_{{\mathbb {F}}}$. Equations (1) and (2) imply that $\dim ({\mathcal {S}}_{\text {FMSDR}})={\text {rank}}(\mathbf {B}) \le p$.

The first *model-based* SDR method for the estimation of ${\mathcal {S}}_{\text {FMSDR}}$ in (1), *principal fitted components* (PFC [12]), requires $\mathbf {X}$ follow model (2) and also is conditionally normally distributed given *Y*, with

5 $$\begin{aligned} \mathbf {X}=\varvec{\mu }+\varvec{\varGamma }\varvec{\gamma }\mathbf {f}_Y +\varvec{\varepsilon }, \quad \varvec{\varepsilon }\sim N_p(\mathbf {0}, \varvec{\varDelta }), \end{aligned}$$

where $\varvec{\varGamma }\in {{\mathbb {R}}}^{p\times d}$ is an orthogonal basis of the linear space ${\mathcal {S}}_{{\varvec{\scriptstyle \varGamma }}}= {\text {span}}\left\{ \varvec{\mu }_Y-\varvec{\mu }, Y \in S_Y\right\}$, with $S_Y$ the sample space of *Y*, and $\varvec{\gamma }\in {{\mathbb {R}}}^{d\times r}$ an unrestricted rank *d* parameter matrix, with $d\le r$. Thus, PFC is a constrained version of PIR [3] in that it also requires $\mathbf {X}\mid Y$ be normal with constant variance $\varvec{\varDelta }$, and the rank *d* of $\mathbf {B}$ in (2) be known so that $\mathbf {B}=\varvec{\varGamma }\varvec{\gamma }$.

Under (5), [12] showed ${\mathcal {S}}_{\text {FMSDR}}={\text {span}}(\varvec{\varGamma })$ and derived the maximum likelihood estimate (MLE) of ${\mathcal {S}}_{\text {FMSDR}}$ to be

6 $$\begin{aligned} {\widehat{{\mathcal {S}}}}_{\text {FMSDR}}&= {\widehat{\varvec{\varSigma }}}_{\mathbf {x}}^{-1}{\widehat{{\mathcal {S}}}}_{{\varvec{\scriptstyle \varGamma }}}={\widehat{\varvec{\varDelta }}}^{-1}{\widehat{{\mathcal {S}}}}_{{\varvec{\scriptstyle \varGamma }}} \nonumber \\&= {\widehat{\varvec{\varDelta }}}_{\text {MLE}}^{-1/2}{\text {span}}_d({\widehat{\varvec{\varDelta }}}_{\text {MLE}}^{-1/2}{\widehat{\varvec{\varDelta }}}_{\text {fit}}{\widehat{\varvec{\varDelta }}}_{\text {MLE}}^{-1/2}), \end{aligned}$$

where

7 $$\begin{aligned} {\widehat{\varvec{\varDelta }}}_{\text {MLE}}&= {\widehat{\varvec{\varDelta }}}_{\text {res}} + {\widehat{\varvec{\varDelta }}}_{\text {res}}^{1/2} {\widehat{\mathbf {V}}} {\widehat{\mathbf {K}}} {\widehat{\mathbf {V}}}^T {\widehat{\varvec{\varDelta }}}_{\text {res}}^{1/2} . \end{aligned}$$

In (7), ${\widehat{\varvec{\varDelta }}}_{\text {res}}$ is obtained by multiplying (4) by $(n-{\text {rank}}({\mathbb {F}}))/n$, and ${\widehat{\varvec{\varDelta }}}_{\text {fit}}= {\mathbb {X}}^T {\mathbb {X}}/n- {\widehat{\varvec{\varDelta }}}_{\text {res}}={\mathbb {X}}^T {\mathbb {X}}/n-{\mathbb {X}}^T \mathbf {Q}_{{\mathbb {F}}} {\mathbb {X}}/n= {\mathbb {X}}^T \mathbf {P}_{{\mathbb {F}}} {\mathbb {X}}/n$. The eigenvectors of ${\widehat{\varvec{\varDelta }}}_{\text {res}}^{-1/2}{\widehat{\varvec{\varDelta }}}_{\text {fit}}{\widehat{\varvec{\varDelta }}}_{\text {res}}^{-1/2}$ are the columns of ${\widehat{\mathbf {V}}}=({\widehat{\mathbf {v}}}_1,\ldots , {\widehat{\mathbf {v}}}_p)$ that correspond to its ordered eigenvalues, ${\hat{\lambda }}_1 \ge \ldots \ge {\hat{\lambda }}_d > {\hat{\lambda }}_{d+1} \ge \ldots \ge {\hat{\lambda }}_p$, and

$$\begin{aligned} {\widehat{\mathbf {K}}} ={\text {diag}}(0,\ldots ,0,{{\hat{\lambda }}}_{d+1},\ldots ,\hat{\lambda }_p).\end{aligned}$$

When $d=r$, (7) reduces to ${\widehat{\varvec{\varDelta }}}_{\text {MLE}} = {\widehat{\varvec{\varDelta }}}_{\text {res}}.$ The MLE of the sufficient reduction is

8 $$\begin{aligned} {\widehat{\mathbf {R}}}_{\text {MLE}}(\mathbf {X}) = \left( {\widehat{\mathbf {v}}}_1^T{\widehat{\varvec{\varDelta }}}_{\text {MLE}}^{-1/2} \mathbf {X}, \ldots , {\widehat{\mathbf {v}}}_d^T {\widehat{\varvec{\varDelta }}}_{\text {MLE}}^{-1/2}\mathbf {X}\right) . \end{aligned}$$

## Matrix-valued predictors

For ease of exposition, we present the model in the longitudinal setting, where the $p \times 1$ predictor vector $\mathbf {X}$ is measured at *T* different time points. Specifically, for sample *i* with response variable $Y_i \in {{\mathbb {R}}}$, $i=1,\ldots ,n$, the predictors can be represented as the $p \times T$-matrix

9 $$\begin{aligned} \mathbf {X}_i = (\mathbf {X}_{i1},\ldots ,\mathbf {X}_{iT})&= \begin{bmatrix} X_{i11} &{} \cdots &{} X_{i1T}\\ X_{i21} &{} \cdots &{} X_{i2T}\\ \vdots &{} \ddots &{} \vdots \\ X_{ip1} &{} \cdots &{} X_{ipT} \end{bmatrix}, \end{aligned}$$

which corresponds to the $pT \times 1$ $\text {vec}(\mathbf {X}_{i})=(\mathbf {X}_{i1}^T,\ldots ,\mathbf {X}_{iT}^T)^T$, comprised of the columns of $\mathbf {X}_i$ in (9) stacked one after another. We assume that all samples have measurements for all predictors at the same time points.

To accommodate the longitudinal structure of $\mathbf {X}\mid Y$, we assume that the centered first moment of $\mathbf {X}$ is decomposed into a time and a predictor component as in [34], and write the linear inverse regression model (2) as bilinear in the rows and columns of $\mathbf {X}$,

10 $$\begin{aligned} \mathbf {X}&= \varvec{\mu }+ \varvec{\beta }\mathbf {f}_Y \varvec{\alpha }^T+ \varvec{\varepsilon }, \end{aligned}$$

where $\mathbf {f}_Y$ is a $k \times r$ matrix of functions in *Y* with ${\mathbb {E}}( \mathbf {f}_Y) =0$, $\varvec{\alpha }\in {\mathbb {R}}^{T\times r}$, and $\varvec{\beta }\in {{\mathbb {R}}}^{p \times k}$. In vector form, model (10) is written as

11 $$\begin{aligned} \text {vec}( \mathbf {X})&= \text {vec}(\varvec{\mu }) + (\varvec{\alpha }\otimes \varvec{\beta }) \text {vec}(\mathbf {f}_y) + \text {vec}( \varvec{\varepsilon }). \end{aligned}$$

The $T \times r$ parameter matrix $\varvec{\alpha }$ captures the mean structure over time, and the $p \times k$ matrix $\varvec{\beta }$ captures the mean structure of the predictors regardless of time. The error $\varvec{\varepsilon }$ satisfies ${\mathbb {E}}(\varvec{\varepsilon })=0$ and ${\mathbb {V}}\text {ar}(\varvec{\varepsilon }\mid Y)={\mathbb {V}}\text {ar}(\mathbf {X}\mid Y)=\varvec{\varDelta }_Y$. Model (11) is analogous to model (2) with the difference that $\text {vec}(\mathbf {f}_y)$ in (11) is a $kr \times 1$-vector and the parameter matrix $\mathbf {B}$ is replaced by the Kronecker product of $\varvec{\alpha }$ and $\varvec{\beta }$, which induces sparsity in the sense of reducing the number of parameters to estimate.^2^

[34] showed that, letting $\varvec{\varSigma }_{\mathbf {x}}$ denote the $pT \times pT$ covariance matrix of $\text {vec}(\mathbf {X})$, and $\varvec{\varDelta }={\mathbb {E}}(\varvec{\varDelta }_Y)$,

12 $$\begin{aligned} {\mathcal {S}}_{\text {FMSDR}}&= \varvec{\varSigma }_{\mathbf {x}}^{-1} {\text {span}}\left( \varvec{\alpha }\otimes \varvec{\beta }\right) = \varvec{\varDelta }^{-1} {\text {span}}\left( \varvec{\alpha }\otimes \varvec{\beta }\right) , \end{aligned}$$

with dimension $$\dim ({\mathcal {S}}_{\text {FMSDR}})={\text {rank}}(\varvec{\alpha }) {\text {rank}}(\varvec{\beta })$$.

For the PFC version of model (11), we use the corresponding parameterization of the two parameter matrices $\varvec{\alpha }\in {{\mathbb {R}}}^{T \times r}$ and $\varvec{\beta }\in {\mathbb {R}}^{p\times k}$, which are both unconstrained. Assuming ${\text {rank}}(\varvec{\alpha })=d_1$ and ${\text {rank}}(\varvec{\beta })=d_2$, we let $\varvec{\alpha }= \varvec{\varGamma }_1 \varvec{\gamma }_1$, where $\varvec{\varGamma }_1$ is a $T \times d_1$ semi-orthogonal matrix whose columns form a basis for the $d_1$-dimensional ${\text {span}}(\varvec{\alpha })$, and $\varvec{\gamma }_1$ is an unconstrained $d_1 \times r$ matrix of rank $d_1$. Similarly, there exists a $p \times d_2$ semi-orthogonal matrix $\varvec{\varGamma }_2$ whose columns form a basis for the $d_2$-dimensional subspace ${\text {span}}(\varvec{\beta })$, and a $d_2 \times k$ rank $d_2$ unconstrained matrix $\varvec{\gamma }_2$, so that $\varvec{\beta }= \varvec{\varGamma }_2 \varvec{\gamma }_2$. Using this parameterization, model (11) becomes

13 $$\begin{aligned} \text {vec}(\mathbf {X}- \varvec{\mu })&= ( \varvec{\varGamma }_1 \varvec{\gamma }_1 \otimes \varvec{\varGamma }_2 \varvec{\gamma }_2) \text {vec}(\mathbf {f}_Y) + \text {vec}(\varvec{\varepsilon }) \nonumber \\&= (\varvec{\varGamma }_1 \otimes \varvec{\varGamma }_2) (\varvec{\gamma }_1 \otimes \varvec{\gamma }_2) \text {vec}(\mathbf {f}_y) + \text {vec}(\varvec{\varepsilon }). \end{aligned}$$

It readily follows that ${\text {span}}\left( \varvec{\mu }_Y -\varvec{\mu }\right) ={\text {span}}(\varvec{\varGamma }_1 \otimes \varvec{\varGamma }_2)$, with $\dim \left( {\text {span}}\left( \varvec{\mu }_Y -\varvec{\mu }\right) \right) ={\text {rank}}(\varvec{\varGamma }_1 \otimes \varvec{\varGamma }_2)=d_1 d_2$. As a consequence, (12) yields

14 $$\begin{aligned} {\mathcal {S}}_{\text {FMSDR}}&=\varvec{\varSigma }_{\mathbf {x}}^{-1} {\mathcal {S}}_{{\varvec{\scriptstyle \varGamma }}_1 \otimes {\varvec{\scriptstyle \varGamma }}_2}=\varvec{\varDelta }^{-1} {\mathcal {S}}_{{\varvec{\scriptstyle \varGamma }}_1 \otimes {\varvec{\scriptstyle \varGamma }}_2} \end{aligned}$$

with $\dim ({\mathcal {S}}_{\text {FMSDR}})=d_1d_2$. When $\varvec{\varSigma }_{\mathbf {x}}$ is separable; i.e., $\varvec{\varSigma }_{\mathbf {x}}= \varvec{\varSigma }_1 \otimes \varvec{\varSigma }_2$, or, slightly less restrictive, when $\varvec{\varDelta }_y={\mathbb {V}}\text {ar}(\text {vec}(\mathbf {X})\mid Y=y) = \varvec{\varDelta }_{1y} \otimes \varvec{\varDelta }_{2y}$, then

$$\begin{aligned} {\mathcal {S}}_{\text {FMSDR}}&={\mathcal {S}}_{{\varvec{\scriptstyle \varSigma }}_1^{-1}{\varvec{\scriptstyle \varGamma }}_1 \otimes {\varvec{\scriptstyle \varSigma }}_2^{-1}{\varvec{\scriptstyle \varGamma }}_2} ={\mathcal {S}}_{ \varvec{\scriptstyle {\varDelta }}_1^{-1}{\varvec{\scriptstyle \varGamma }}_1 \otimes \varvec{\scriptstyle {\varDelta }}_2^{-1}{\varvec{\scriptstyle \varGamma }}_2} \end{aligned}$$

since $\varvec{\varDelta }= {\mathbb {E}}\left( {\mathbb {V}}\text {ar}(\mathbf {X}\mid Y) \right) = \varvec{\varDelta }_1 \otimes \varvec{\varDelta }_2$. In this case, the number of parameters that are needed to estimate ${\mathcal {S}}_{\text {FMSDR}}$ in (14) is further reduced.

## Estimating ${\mathcal {S}}_{\text {FMSDR}}$ using matrix-valued predictors

We propose several approaches to estimate ${\mathcal {S}}_{\text {FMSDR}}$ in (14) by estimating the component matrices $\varvec{\alpha }$ and $\varvec{\beta }$ and $\varvec{\varGamma }_1$ and $\varvec{\varGamma }_2$ in models (11) and (13). We assume that the dimension *d* is known and comment on inference on *d* for all approaches in Sect. 8.

### Least squares Kronecker parametric inverse regression, (K-PIR (ls))

To obtain least squares (ls)-based estimates of ${\mathcal {S}}_{\text {FMSDR}}$ under model (11), we assume that the predictors are centered around their overall mean $\varvec{\mu }$. Using the sample level notation defined in Sect. 2.1 and letting ${\widetilde{\mathbf {X}}}_i =\mathbf {X}_{i} - {\overline{\mathbf {X}}}$, $i=1,\ldots ,n$, the model becomes

15 $$\begin{aligned} {\mathbb {X}}&= {\mathbb {F}}_{y} (\varvec{\alpha }\otimes \varvec{\beta })^T + \varvec{\varepsilon }, \end{aligned}$$

where ${\mathbb {X}}_{y}: n \times pT$ with *i*th row $\text {vec}({\widetilde{\mathbf {X}}}_{i})$, $\varvec{\alpha }\in {{\mathbb {R}}}^{T \times r}$, $\varvec{\beta }\in {\mathbb {R}}^{p\times k}$, $\mathbb {\varvec{\varepsilon }}: n \times pT$ with ${\mathbb {E}}(\mathbb {\varvec{\varepsilon }})=\mathbf {0}$, ${\mathbb {V}}\text {ar}\left( \text {vec}(\mathbb {\varvec{\varepsilon }})\right) = \varvec{\varDelta }\otimes \mathbf {I}_n$, and ${\mathbb {F}}_y$ is an $n \times kr$ matrix with entries ${\widetilde{\mathbf {f}}}_{y_i}$, where ${\widetilde{\mathbf {f}}}_{y_i}=\text {vec}(\mathbf {f}_{y_i})- {{\bar{\mathbf {f}}}}_y$, and ${{\bar{\mathbf {f}}}}_y$ is the $kr \times 1$ empirical mean of $\text {vec}(\mathbf {f}_{y_i})$, $i=1,\ldots ,n$.

The following theorem, proved in “Appendix,” summarizes the approach and properties of the resulting estimates.

#### Theorem 1

Assume the data ${\mathbb {X}}$ follow model (15). Let ${\widehat{\mathbf {B}}}= ({\mathbb {F}}_y^T{\mathbb {F}}_y)^{-1}{\mathbb {F}}_y^T {\mathbb {X}}$ denote the ordinary least squares estimate in the unconstrained model ${\mathbb {X}}= {\mathbb {F}}_{y} \mathbf {B}+ \varvec{\varepsilon }$. The matrices ${\widehat{\varvec{\alpha }}}$ and ${\widehat{\varvec{\beta }}}$ defined as

16 $$\begin{aligned} ({\widehat{\varvec{\alpha }}}, {\widehat{\varvec{\beta }}})&= {\text {argmin}}_{\varvec{\alpha }, \varvec{\beta }} \Vert {\widehat{\mathbf {B}}}^T - \varvec{\alpha }\otimes \varvec{\beta }\Vert ^2 \end{aligned}$$

and estimated using algorithm 2 in [40], converge in probability to $\varvec{\alpha }$ and $\varvec{\beta }$ in the constrained model (11).^3^ That is,

17 $$\begin{aligned} {\widehat{\varvec{\alpha }}} \otimes {\widehat{\varvec{\beta }}} \overset{p}{\rightarrow } \varvec{\alpha }\otimes \varvec{\beta }. \end{aligned}$$

When the distribution of $\mathbf {X}\mid Y$ belongs to the exponential family, then ${\widehat{\varvec{\alpha }}}$ and ${\widehat{\varvec{\beta }}}$ are asymptotically normal.

We refer to any matrices that are obtained as solutions to (16) as *VLP* (Van Loan and Pitsianis [40]) approximations. The algorithm is described in “Appendix.”

Given ${{\widehat{\varvec{\alpha }}}}$ and ${{\widehat{\varvec{\beta }}}}$, the least squares-based estimate of $\varvec{\varDelta }={\mathbb {V}}\text {ar}(\varvec{\varepsilon }\mid Y)$ is

18 $$\begin{aligned} {\widehat{\varvec{\varDelta }}}_{\text {ls}}&= \frac{1}{n-{\text {rank}}({\mathbb {F}}_{y})} \sum _i^n \left( \text {vec}({\widetilde{\mathbf {X}}}_{i}) -({\widehat{\varvec{\alpha }}} \otimes {\widehat{\varvec{\beta }}} ) {\widetilde{\mathbf {f}}}_{y_i} \right) \nonumber \\&\qquad \qquad \qquad \qquad \left( \text {vec}({\widetilde{\mathbf {X}}}_{i}) - ({\widehat{\varvec{\alpha }}} \otimes {\widehat{\varvec{\beta }}} ) {\widetilde{\mathbf {f}}}_{y_i}\right) ^T. \end{aligned}$$

### ML Kronecker parametric inverse regression (K-PIR (mle))

We derive the MLEs for $\varvec{\alpha }$ and $\varvec{\beta }$ in model (11) under the additional assumption that $\mathbf {X}_i\mid (Y=y_i)$, $i=1,\ldots ,n$, are normally distributed,

19 $$\begin{aligned} \text {vec}(\mathbf {X}_{i}) \sim N_{pT}( \text {vec}(\varvec{\mu })+(\varvec{\alpha }\otimes \varvec{\beta }) \text {vec}({\tilde{\mathbf {f}}}_{y_i}), \varvec{\varDelta }), \end{aligned}$$

where ${\widetilde{\mathbf {f}}}_{y_i}$ is defined (Eq. (15)). The corresponding log-likelihood is

20 $$\begin{aligned}&l(\varvec{\mu }, \varvec{\alpha }, \varvec{\beta }, \varvec{\varDelta })= -\frac{nTp}{2} \log (2 \pi ) - \frac{n}{2} \log |\varvec{\varDelta }| \nonumber \\&\quad -\frac{1}{2} \sum _{i=1}^n \left( \text {vec}( \mathbf {X}_{i}) -\text {vec}(\varvec{\mu })- (\varvec{\alpha }\otimes \varvec{\beta }){\widetilde{\mathbf {f}}}_{y_i} \right) ^T \nonumber \\&\quad \varvec{\varDelta }^{-1} \left( \text {vec}( \mathbf {X}_{i}) -\text {vec}(\varvec{\mu }) -(\varvec{\alpha }\otimes \varvec{\beta }) {\widetilde{\mathbf {f}}}_{y_i} \right) , \end{aligned}$$

The MLE of $\varvec{\mu }$ when the other parameters are fixed is the sample mean ${\overline{\mathbf {X}}}$. We substitute ${\overline{\mathbf {X}}}$ for $\varvec{\mu }$ and use the centered observations ${\widetilde{\mathbf {X}}}_{i} =\mathbf {X}_{i} - {\overline{\mathbf {X}}}$ in what follows. For fixed $\varvec{\alpha }$ and $\varvec{\beta }$, solving the corresponding score equation for $\varvec{\varDelta }$ yields

21 $$\begin{aligned} {\widehat{\varvec{\varDelta }}}&= \frac{1}{n} \sum _i^n \left( \text {vec}({\widetilde{\mathbf {X}}}_{i}) - ( {\varvec{\alpha }} \otimes {\varvec{\beta }}){\widetilde{\mathbf {f}}}_{y_i}) \right) \nonumber \\&\qquad \left( \text {vec}({\widetilde{\mathbf {X}}}_{i}) - ({\varvec{\alpha }} \otimes {\varvec{\beta }}) {\widetilde{\mathbf {f}}}_{y_i}\right) ^T. \end{aligned}$$

The score equations for $\varvec{\alpha }$ and $\varvec{\beta }$, however, do not yield closed form solutions, and we employ the following iterative algorithm for estimation.

*K-PIR MLE Algorithm:*

1. Initialize ${\widehat{\varvec{\varDelta }}}$ at the value ${\widehat{\varvec{\varDelta }}}_0$ from least squares in (18).
2. Compute ${\widehat{\varvec{\alpha }}}_1$, ${\widehat{\varvec{\beta }}}_1$ by optimizing the log-likelihood in (20) numerically with starting values $({\widehat{\varvec{\alpha }}}^0,{\widehat{\varvec{\beta }}}^0) = ({\widehat{\varvec{\alpha }}}_{\text {ls}},{\widehat{\varvec{\beta }}}_{\text {ls}})$, where $({\widehat{\varvec{\alpha }}}_{\text {ls}},{\widehat{\varvec{\beta }}}_{\text {ls}})$ is the approximate ls solution computed from (16).
3. Compute ${\widehat{\varvec{\varDelta }}}_1$ from (21) with $\varvec{\alpha }={\widehat{\varvec{\alpha }}}_1$ and $\varvec{\beta }={\widehat{\varvec{\beta }}}_1$.
4. Repeat steps 2 and 3 until $\Vert {{\widehat{\varvec{\varDelta }}}}_i - {{\widehat{\varvec{\varDelta }}}}_{i+1}\Vert /\Vert {{\widehat{\varvec{\varDelta }}}}_i\Vert < \epsilon _1$ and $\Vert ({\widehat{\varvec{\alpha }}}_i \otimes {\widehat{\varvec{\beta }}}_i) - ({\widehat{\varvec{\alpha }}}_{i+1} \otimes {\widehat{\varvec{\beta }}}_{i+1})\Vert /\Vert {\widehat{\varvec{\alpha }}}_i \otimes {\widehat{\varvec{\beta }}}_i\Vert < \epsilon _2$, for some small $\epsilon _1>0$ and $\epsilon _2>0$.

We estimate ${\mathcal {S}}_{\text {FMSDR}}$ in (12), assuming that $d_1$ and $d_2$ are known, with

22 $$\begin{aligned} {\widehat{{\mathcal {S}}}}_{\text {FMSDR}}&={\widehat{\varvec{\varDelta }}}^{-1} \left( {\widehat{\varvec{\varGamma }}}_1 \otimes {\widehat{\varvec{\varGamma }}}_2 \right) , \end{aligned}$$

where ${\widehat{\varvec{\varGamma }}}_1$ and ${\widehat{\varvec{\varGamma }}}_2$ are the first $d_1$ and $d_2$ singular vectors of ${\widehat{\varvec{\alpha }}}$ and ${\widehat{\varvec{\beta }}}$, respectively.

### Kronecker principal fitted components (K-PFC)

The log-likelihood under model (13) with $\varvec{\varepsilon }\sim N_{pT}(\mathbf {0},\varvec{\varDelta })$ has a different mean structure from (20), which is

23 $$\begin{aligned}&l(\varvec{\mu }, {\mathcal {S}}_{{\varvec{\scriptstyle \varGamma }}_1 \otimes {\varvec{\scriptstyle \varGamma }}_2}, \varvec{\gamma }_1 \otimes \varvec{\gamma }_2, \varvec{\varDelta }) \nonumber \\&\quad = -\frac{npT}{2} \log (2\pi ) - \frac{n}{2}\log |\varvec{\varDelta }| \nonumber \\&\qquad -\frac{1}{2}\sum _i \left( \text {vec}(\mathbf {X}_i)-\text {vec}(\varvec{\mu }) - (\varvec{\varGamma }_1 \otimes \varvec{\varGamma }_2) (\varvec{\gamma }_1 \otimes \varvec{\gamma }_2){\widetilde{\mathbf {f}}}_{y_i}\right) ^T \nonumber \\&\varvec{\varDelta }^{-1} \left( \text {vec}(\mathbf {X}_i)-\text {vec}(\varvec{\mu }) - (\varvec{\varGamma }_1 \otimes \varvec{\varGamma }_2) (\varvec{\gamma }_1 \otimes \varvec{\gamma }_2){\widetilde{\mathbf {f}}}_{y_i}\right) . \end{aligned}$$

Let $\varvec{\varGamma }=\varvec{\varGamma }_1 \otimes \varvec{\varGamma }_2$ and $\varvec{\gamma }=\varvec{\gamma }_1 \otimes \varvec{\gamma }_2$. Then, $\varvec{\varGamma }$ is a $pT \times d_1d_2$ semi-orthogonal matrix of rank $d=d_1 d_2$, and $\varvec{\gamma }$ is a $d \times kr$ matrix of rank *d*, but otherwise unconstrained. [12] computed the MLEs of $\varvec{\mu }$, $\varvec{\varGamma }$, and $\varvec{\gamma }$ in model (5) with $\mathbf {B}^T=\varvec{\varGamma }\varvec{\gamma }$ to be

24 $$\begin{aligned} \text {vec}({\widehat{\varvec{\mu }}})&= {\overline{\mathbf {X}}} \end{aligned}$$

25 $$\begin{aligned} {\widehat{{\mathcal {S}}}}_{{\varvec{\scriptstyle \varGamma }}}&= {\widehat{\varvec{\varDelta }}}_{\text {MLE}}^{1/2}{\text {span}}_d({\widehat{\varvec{\varDelta }}}_{\text {MLE}}^{-1/2} {\widehat{\varvec{\varDelta }}}_{\text {fit}}{\widehat{\varvec{\varDelta }}}_{\text {MLE}}^{-1/2}) \end{aligned}$$

26 $$\begin{aligned} {\widehat{\varvec{\gamma }}}&= \left( {\widehat{\varvec{\varGamma }}}^T {\widehat{\varvec{\varDelta }}}_{\text {MLE}}^{-1}{\widehat{\varvec{\varGamma }}}\right) ^{-1} {\widehat{\varvec{\varGamma }}}^T{\widehat{\varvec{\varDelta }}}_{\text {MLE}}^{-1} {\widehat{\mathbf {B}}}^T, \end{aligned}$$

where ${\widehat{\mathbf {B}}}^T={\mathbb {X}}^T {\mathbb {F}}_y ({\mathbb {F}}_y^T{\mathbb {F}}_y)^{-1}$ is the OLS for the unconstrained model ${\mathbb {X}}= {\mathbb {F}}_{y} \mathbf {B}+ \mathbb {\varvec{\varepsilon }}$, ${\widehat{\varvec{\varGamma }}}$ is any orthonormal basis for ${\widehat{{\mathcal {S}}}}_{{\varvec{\scriptstyle \varGamma }}}$ and ${\text {span}}_d({\widehat{\varvec{\varDelta }}}_{\text {MLE}}^{-1/2} {\widehat{\varvec{\varDelta }}}_{\text {fit}}{\widehat{\varvec{\varDelta }}}_{\text {MLE}}^{-1/2})$ denotes the span of the first *d* eigenvectors of ${\widehat{\varvec{\varDelta }}}_{\text {MLE}}^{-1/2}{\widehat{\varvec{\varDelta }}}_{\text {fit}} {\widehat{\varvec{\varDelta }}}_{\text {MLE}}^{-1/2}$, with

27 $$\begin{aligned} {\widehat{\varvec{\varDelta }}}_{\text {fit}}&={\mathbb {X}}\mathbf {P}_{{\mathbb {F}}_y}{\mathbb {X}}/n , \end{aligned}$$

and $\mathbf {P}_{{\mathbb {F}}}={\mathbb {F}}_y^T({\mathbb {F}}_y^T{\mathbb {F}}_y)^{1}{\mathbb {F}}_y$. We show in “Appendix” that the Kronecker product structure constraint on the parameter matrix $\mathbf {B}=\varvec{\alpha }^T \otimes \varvec{\beta }^T$ does not alter the formulae for the MLEs until the last step. That is,

28 $$\begin{aligned} {\widehat{{\mathcal {S}}}}_{{\varvec{\scriptstyle \varGamma }}}&= {\widehat{{\mathcal {S}}}}_{{\varvec{\scriptstyle \varGamma }}_1 \otimes {\varvec{\scriptstyle \varGamma }}_2} \end{aligned}$$

29 $$\begin{aligned} {\widehat{\varvec{\gamma }}}&={\widehat{\varvec{\gamma }}}_1 \otimes {\widehat{\varvec{\gamma }}}_2 =\left( ({\widehat{\varvec{\varGamma }}}_1 \otimes {\widehat{\varvec{\varGamma }}}_2)^T {\widehat{\varvec{\varDelta }}}_{\text {MLE}}^{-1} ({\widehat{\varvec{\varGamma }}}_1 \otimes {\widehat{\varvec{\varGamma }}}_2)\right) ^{-1} \nonumber \\&\qquad ({\widehat{\varvec{\varGamma }}}_1 \otimes {\widehat{\varvec{\varGamma }}}_2)^T {\widehat{\varvec{\varDelta }}}_{\text {MLE}}^{-1}{\widehat{\mathbf {B}}}^T. \end{aligned}$$

The expression for ${\widehat{\varvec{\varDelta }}}_{\text {MLE}}$ is given in equation (7). In the full-rank setting, i.e., when $d_1=r$ and $d_2=k$, (7) simplifies to ${\widehat{\varvec{\varDelta }}}_{\text {MLE}}={\widehat{\varvec{\varDelta }}}_{\text {res}}$, since ${\widehat{\mathbf {K}}}$ is then a matrix of zeros.

#### Remark 1

In the standard MLE approach of Sect. 4.2, the number of unknown parameters in $\varvec{\alpha }$ and $\varvec{\beta }$ is $Tr+pk$, whereas in the PFC parameterization is $Td_1+pd_2$, which can be significantly smaller in the non-full-rank setting where $d_1<r$ and $d_2<k$.

*K-PFC Least Squares Estimation Algorithms:*

We propose several algorithms utilizing the VLP approximation for estimating ${\mathcal {S}}_{\text {FMSDR}}$ under model (15) and the additional assumption that $\varvec{\varepsilon }\sim N_{pT}(\mathbf {0},\varvec{\varDelta })$.

1. Compute ${\widehat{\mathbf {B}}}^T={\mathbb {X}}^T{\mathbb {F}}_y({\mathbb {F}}_y^T{\mathbb {F}}_y)^{-1}$, ${\widehat{\varvec{\varDelta }}}_{\text {fit}}={\mathbb {X}}^T\mathbf {P}_{{\mathbb {F}}_y}{\mathbb {X}}/n$, and ${\widehat{\varvec{\varDelta }}}_{\text {res}}= {\widehat{\varvec{\varDelta }}} -{\widehat{\varvec{\varDelta }}}_{\text {fit}}$, where ${\widehat{\varvec{\varDelta }}}={\mathbb {X}}^T{\mathbb {X}}/n$.
2. Compute $${\widehat{\varvec{\varDelta }}}_{\text {MLE}}$$ from (7).
3. Set ${\widehat{\varvec{\varGamma }}}$ to be the first *d* eigenvectors of (25).
4. Estimate $${\widehat{\varvec{\gamma }}}$$
  - using expression (26) and ${\widehat{\mathbf {B}}}^T={\widehat{\varvec{\varGamma }}}{\widehat{\varvec{\gamma }}}$. Compute ${\widehat{\varvec{\alpha }}}$ and ${\widehat{\varvec{\beta }}}$ by applying the VLP approximation (K-PFC1).
  - applying VLP to ${\widehat{\varvec{\varGamma }}}$ to obtain ${\widehat{\varvec{\varGamma }}}_1$ and ${\widehat{\varvec{\varGamma }}}_2$, and then compute ${\widehat{\varvec{\gamma }}}$ from (29).
    4bi. Compute $${\widehat{\varvec{\alpha }}} \otimes {\widehat{\varvec{\beta }}} = ({\widehat{\varvec{\varGamma }}}_1\otimes {\widehat{\varvec{\varGamma }}}_2) {\widehat{\varvec{\gamma }}}$$   (K-PFC2).
    4bii. Apply VLP to ${\widehat{\varvec{\gamma }}}$ to obtain ${\widehat{\varvec{\gamma }}}_1$ and ${\widehat{\varvec{\gamma }}}_2$ and then calculate ${\widehat{\varvec{\alpha }}} = {\widehat{\varvec{\varGamma }}}_1{\widehat{\varvec{\gamma }}}_1$ and ${\widehat{\varvec{\beta }}} = {\widehat{\varvec{\varGamma }}}_2 {\widehat{\varvec{\gamma }}}_2$ (K-PFC3).

#### Remark 2

K-PIR (ls) in Sect. 4.1 is based on model (11) without assuming a specific distribution for the inverse predictors, $\mathbf {X}\mid Y$. K-PIR (mle) in Sect. 4.2 also uses model (11), but requires $\mathbf {X}\mid Y$ be normal as in (19). The three K-PFC methods use model (13) under the assumption of normality of $\mathbf {X}\mid Y$. K-PIR (ls) and K-PFC1, K-PFC2, K-PFC3 estimate (14) using the Van Loan and Pitsianis (VLP) [40] least squares approximation algorithm applied to different parameter matrices.

**Fig. 1:**
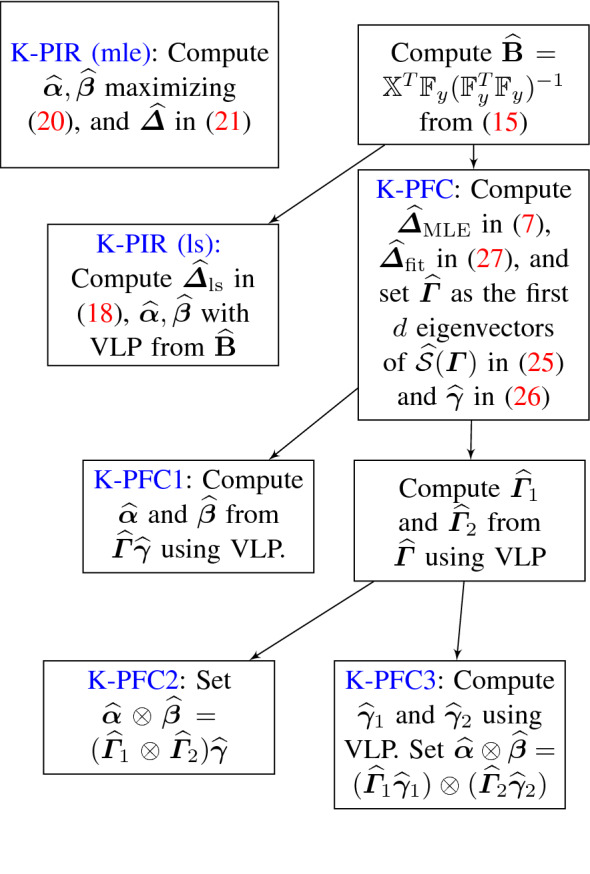
Flowchart of K-PIR and K-PFC Algorithms

### Variable selection: sparse K-PIR and K-PFC

In addition to reducing the dimension of the predictors, it is desirable to identify those associated with the outcome and remove irrelevant and redundant ones when computing sufficient reductions. We adapt results of [7], a version of group lasso [6], to the Kronecker product setting.

One can easily show that the coordinate-independent sparse sufficient dimension reduction estimator (CISE) of ${\mathcal {S}}_{\text {FMSDR}}$ in (14) is ${\widehat{{\mathcal {S}}}}_{\text {FMSDR}(\text {CISE})}={\text {span}}({\widehat{\varvec{\varSigma }}}_{\mathbf {x}}^{-1/2}{\widetilde{\varvec{\varGamma }}})$ with

30 $$\begin{aligned} {\widetilde{\varvec{\varGamma }}}&= {\text {argmin}}_{\varvec{\varGamma }} J_d(\varvec{\varGamma }) \quad \text{ subject } \text{ to } \varvec{\varGamma }^T \varvec{\varGamma }= \mathbf {I}_d, \end{aligned}$$

where

31 $$\begin{aligned} J_d(\varvec{\varGamma })&= -\text {tr}(\varvec{\varGamma }^T{\widehat{\varvec{\varSigma }}}_{\mathbf {x}}^{-1/2} {\widehat{\varvec{\varDelta }}}_{\text {fit}}{\widehat{\varvec{\varSigma }}}_{\mathbf {x}}^{-1/2}\varvec{\varGamma }) \nonumber \\&\qquad + \lambda \sum _{i=1}^p \Vert {\widehat{\varvec{\varSigma }}}_{\mathbf {x}}^{-1/2} \varvec{\varGamma }_i^T \Vert _2, \end{aligned}$$

$\varvec{\varGamma }_i^T$ is the *i*th row of $\varvec{\varGamma }=\varvec{\varGamma }_1 \otimes \varvec{\varGamma }_2$, $\lambda \ge 0$ is a regularization parameter, ${\widehat{\varvec{\varDelta }}}_{\text {fit}}$ is given in (27), and $\Vert \,.\,\Vert _2$ denotes the $L_2$ norm.

The minimization of (30) is a Grassmann manifold optimization problem. Since $\Vert \cdot \Vert _2$ is non-differentiable at zero, traditional Grassmann manifold optimization techniques [see [19]] cannot be applied directly. [7] proposed a computational algorithm based on local quadratic approximation [20], and [48] proved that CISE with the BIC-based tuning parameter selection identified the true model consistently, i.e., has the oracle property.

We use the *fast penalized orthogonal iteration* (fast POI) optimization algorithm in [26] to implement CISE. Fast POI is a new algorithm for sparse estimation of eigenvectors in generalized eigenvalue problems, which is much faster and easier to implement than the algorithm in [7]. Fast POI-C, the coordinate-wise version of the algorithm, is guaranteed to converge to the optimal solution [26, 39].

To simultaneously carry out variable selection and dimension reduction in the least squares-based approaches, we first solve (30) to obtain ${\widetilde{\varvec{\varGamma }}}$ and then minimize

32 $$\begin{aligned} \Vert {\widetilde{\varvec{\varGamma }}} - {\widetilde{\varvec{\varGamma }}}_1 \otimes {\widetilde{\varvec{\varGamma }}}_2 \Vert ^2 \end{aligned}$$

via the VLP approximation to find ${\widetilde{\varvec{\varGamma }}}_1$ and ${\widetilde{\varvec{\varGamma }}}_2$. The sparse estimate of the sufficient reduction is

$$\begin{aligned} {\mathcal {S}}_{{{{\hbox {KCISE}}}}}= {\text {span}}\left( {\widehat{\varvec{\varSigma }}}_{\mathbf {x}}^{-1} ({\widetilde{\varvec{\varGamma }}}_1 \otimes {\widetilde{\varvec{\varGamma }}}_2)\right) . \end{aligned}$$

Coordinate-wise SDR selects whole rows (corresponding to particular markers) and whole columns (corresponding to particular time points) separately which are then removed from the model. It does not remove a particular marker only for select time points.

## Simulations

We assessed the performance of K-PIR (ls) in Sect. 4.1, K-PIR (mle) in Sect.  4.2, and the K-PFC least squares algorithms in Sect. 4.3, for estimating the sufficient reduction subspace ${\mathcal {S}}_{\text {FMSDR}}$ using simulations, for both continuous and binary outcomes *Y*.

As mentioned in Introduction, there are very few regression or classification approaches that apply to matrix-valued predictors. The only directly comparable published methods are folded SIR [29] and longitudinal sliced inverse regression (LSIR) [34]. We excluded folded SIR from the simulations due to the instability of its estimation algorithm [see the analysis of the EEG data in Sect. 7]. LSIR [34] assumes both the first and second conditional moments of $\mathbf {X}\mid Y$ have Kronecker product structure; i.e., ${\mathbb {E}}(\mathbf {X}\mid Y)-{\mathbb {E}}(\mathbf {X})= \varvec{\alpha }\otimes \varvec{\beta }$, and ${\mathbb {V}}\text {ar}(\mathbf {X})=\varvec{\varSigma }_{\alpha } \otimes \varvec{\varSigma }_{\beta }$, where $\varvec{\alpha }$ captures the time and $\varvec{\beta }$ the biomarker structure of the predictors. The estimation of the sufficient reduction is based on discretizing the response variable *Y*, if it is not categorical, and using the group sample means to estimate ${\mathcal {S}}_{\text {FMSDR}}$ in (1). LSIR is the Kronecker product version of linear discriminant analysis for matrix-valued data.

For continuous outcomes *Y*, we additionally compared our methods to *(2D)*$^2$ *principal component regression* that we denote as (2D)$^2$PCR, our adaptation of (2D)$^2$PCA [45] and GLRAM [42] to regression with matrix-valued predictors in analogy to principal regression analysis (PCR) [25]. PCR computes linear combinations, principal components (PCs), of vector-valued predictors, using as coefficients the elements of the eigenvectors of the predictor sample covariance matrix arranged with respect to its eigenvalues in decreasing order. We let $\mathbf {U}_{\alpha }= (\mathbf {U}_{1,\alpha },\ldots ,\mathbf {U}_{T,\alpha })$ and $\mathbf {U}_{\beta }= (\mathbf {U}_{1,\beta },\ldots ,\mathbf {U}_{p,\beta })$ denote the *column* and *row* eigenvectors of the $p \times T$ predictor $\mathbf {X}$, respectively. The columns of $\mathbf {U}_{\alpha }$ are the eigenvectors of the $T \times T$ sample *column* covariance matrix ${\widehat{\varvec{\varSigma }}}_{\alpha }=\sum _{j=1}^n (\mathbf {X}_j-{\bar{\mathbf {X}}})^T(\mathbf {X}_j-{\bar{\mathbf {X}}})/n:$, and those of $\mathbf {U}_{\beta }$ are the eigenvectors of the $p \times p$ sample *row* covariance matrix ${\widehat{\varvec{\varSigma }}}_{\beta }=\sum _{j=1}^n (\mathbf {X}_j-{\bar{\mathbf {X}}})(\mathbf {X}_j-{\bar{\mathbf {X}}})^T/n$. We define the (2D)$^2$ PCs of $\mathbf {X}$ to be $\mathbf {X}_i^{\star } = \mathbf {U}_{\beta }^T \mathbf {X}_i \mathbf {U}_{\alpha }$, for $i=1,\ldots , n$, and call the regression of the response *Y* on $\mathbf {X}_i^{\star }$ “(2D)$^2$PCR.”

The (2D)$^2$PCA estimate of $\varvec{\alpha }\otimes \varvec{\beta }$ in (11) is $\mathbf {U}_{\alpha , d_1} \otimes \mathbf {U}_{\beta , d_2}$, where $\mathbf {U}_{\alpha ,d_1}= (\mathbf {U}_{1,\alpha },\ldots ,\mathbf {U}_{d_1,\alpha })$, and $\mathbf {U}_{\beta , d_2}= (\mathbf {U}_{1,\beta },\ldots ,\mathbf {U}_{d_2,\beta })$.

### Estimation of the subspace

#### Data generation for continuous outcome *Y*

To generate data from the model in equation (10), we first generated $y_i \sim N(0,1)$ for $i =1,\ldots ,n$, and then computed the *i*th row $\mathbf {f}_{y_i} = \mathbf {g}_{y_i}-{\bar{\mathbf {g}}}$ of the $n\times rk$ matrix ${\mathbb {F}}_y$, where $\mathbf {g}_{y_i}$ is a vector of Fourier basis functions, $\text {vec}(\mathbf {g}_{y_i}) = (\cos (2\pi {y_i}),\sin (2\pi {y_i}),\ldots ,\cos (2\pi s y_i),\sin (2\pi s y_i) )^T,$ with $2s=rk$. The $n \times pT$ matrix of error terms was generated from the multivariate normal $N_{npT}(\mathbf {0}, \varvec{\varDelta }\otimes \mathbf {I}_n )$, where $\varvec{\varDelta }$ was a positive definite matrix with ones on the diagonal to ensure that all variables have the same scale. We then let $\varvec{\alpha }= \varvec{\varGamma }_1\varvec{\gamma }_1$ and $\varvec{\beta }=\varvec{\varGamma }_2\varvec{\gamma }_2,$ where $\varvec{\varGamma }_1 \in {{\mathbb {R}}}^{T \times d_1}$ and $\varvec{\varGamma }_2 \in {{\mathbb {R}}}^{p \times d_2}$, and computed ${\mathbb {X}}= {\mathbb {F}}_{y} (\varvec{\alpha }\otimes \varvec{\beta })^T + \mathbb {\varvec{\varepsilon }}$, using the parameterization in (15).

We present results for $\varvec{\varGamma }_1$ with entries $[\varvec{\varGamma }_1]_{11}=[\varvec{\varGamma }_1]_{22}=\ldots =[\varvec{\varGamma }_1]_{d_1 d_1}=1$ and zeros elsewhere, and $\varvec{\varGamma }_2$ with entries $[\varvec{\varGamma }_2]_{11}=[\varvec{\varGamma }_2]_{22}=\ldots =[\varvec{\varGamma }_2]_{d_2 d_2}=1$ and zeros elsewhere. The matrices $\varvec{\gamma }_1$ and $\varvec{\gamma }_2$ are $d_1 \times r$ and $d_2 \times k$ matrices of zeros and ones of rank $d_1$ and $d_2$, respectively. The resulting matrices $\varvec{\alpha }$ and $\varvec{\beta }$ also have zeros and ones as entries and are of rank $d_1$ and $d_2$, respectively.

Prior to fitting, we centered the predictors by subtracting their empirical means; i.e., the *i*th row of ${\mathbb {X}}$ was $\mathbf {X}_{i}-{\overline{\mathbf {X}}}$. Therefore, the simulation data follow the model $\text {vec}(\mathbf {X}-\varvec{\mu }) = ( \varvec{\varGamma }_1 \varvec{\gamma }_1 \otimes \varvec{\varGamma }_2 \varvec{\gamma }_2) \text {vec}(\mathbf {f}_y) + \text {vec}(\varvec{\varepsilon })$ in (13).

We let $$p=10, T=8$$ with $$r=k=6$$ for $$d_1=d_2=2,$$ $$d_1=d_2=4,$$ and $$d_1=d_2=6,$$ to assess the impact of the dimension on the estimation procedures. For each setting, we generated 500 data sets of sample sizes $$n=500$$ and $$n=5000 $$ and report means over the 500 repetitions in Tables 1 and 2 .

#### Data generation for binary outcome *Y*

We generated $\mathbf {X}$ from two multivariate normal distributions with equal covariance matrices, $(\mathbf {X}_k\mid Y=i) \sim N( \varvec{\alpha }_i \otimes \varvec{\beta }, \varvec{\varDelta }), k=1,\ldots ,n_i, i=0, 1,$ for $n_0=n_1=n/2,$ for $n=500, 1000$ and $n=2000$ with $p=10$ and $T=5$. Each $\varvec{\alpha }_i, i=0,1,$ was a vector of length *T* and $\varvec{\beta }$ was a vector of length *p*; that is, the dimension is $d=d_1 d_2=1$. We let $\varvec{\beta }=p^{-1/2}(1,\ldots ,1)$ and the entries of $\varvec{\alpha }_0$ be equal to 0, and the entries of $\varvec{\alpha }_1$ were $\varvec{\alpha }_1[k]=(T-k+1)^{-1}$. When *T* denotes time from study baseline, this choice of the $\varvec{\alpha }_1$ coefficients leads to later time points; i.e., measurements more proximal in time to *Y*, contributing more to discrimination of the two groups. The variance matrix of the predictors was separable, $\varvec{\varSigma }_{\mathbf {x}} = {\mathbb {V}}\text {ar}(\mathbf {X})=\varvec{\varSigma }_1 \otimes \varvec{\varSigma }_2$. We imposed an AR(1) structure on both components of $\varvec{\varSigma }_{\mathbf {x}}$; that is, $\text {cor}(X_{ij},X_{ik}) = \rho _T^{|k-j|}$ for $\varvec{\varSigma }_1$, and $\text {cor}(X_{ij},X_{kj} )= \rho _p^{|k-j|}$ for $\varvec{\varSigma }_2$, for various choices of $\rho _T$ and $\rho _p$. The covariance matrix $\varvec{\varDelta }$ was computed using

$$\begin{aligned} \varvec{\varSigma }_{\mathbf {x}}= & {} {\mathbb {E}}( {\mathbb {C}}\text {ov}(\text {vec}(\mathbf {X}) \mid Y)) + {\mathbb {C}}\text {ov}({\mathbb {E}}(\text {vec}(\mathbf {X}) \mid Y)) \\= & {} \varvec{\varDelta }+ {\mathbb {E}}\{ {\mathbb {E}}(\text {vec}(\mathbf {X}) \mid Y){\mathbb {E}}(\text {vec}(\mathbf {X})^T \mid Y)\}\\&\quad -{\mathbb {E}}(\text {vec}(\mathbf {X})) {\mathbb {E}}(\text {vec}(\mathbf {X})^T). \end{aligned}$$

#### Performance evaluation for estimation of the subspace

To evaluate bias, we computed the differences between the estimated and the true matrix values as $E_1=\Vert {\widehat{\varvec{\alpha }}}\otimes {\widehat{\varvec{\beta }}}-\varvec{\alpha }\otimes \varvec{\beta }\Vert / \Vert \varvec{\alpha }\otimes \varvec{\beta }\Vert$ and $E_2=\Vert {\widehat{\varvec{\varDelta }}}-\varvec{\varDelta }\Vert / \Vert \varvec{\varDelta }\Vert$, along with their standard deviations.

**Table 1:** Continuous outcomes *Y* with $$ p = 10, T = 8, r = k = 6$$ and $${\text {rank}}(\varvec{\alpha }) = {\text {rank}}(\varvec{\beta }) = 6$$

Method	Mean $E_1$	SD $E_1$	Mean $E_2$	SD $E_2$	$V_1$	$V_2$	$\Phi$	$\phi _1$	$\phi _2$
$$n = 500$$									
K-PIR (ls)	0.11	0.01	0.32	0.01	0.47	105.40	0.56	0.13	0.19
K-PIR (mle)	0.09	0.01	0.29	0.01	0.17	91.32	0.44	0.10	0.15
K-PFC1	0.11	0.01	0.29	0.01	0.47	79.75	0.56	0.13	0.19
K-PFC2	0.48	0.08	0.29	0.01	6.82	79.75	5.55	1.85	1.86
K-PFC3	0.64	0.18	0.29	0.01	9.50	79.75	5.55	1.85	1.86
(2D)${^2}$PCA	1.41	0.02	86.37	1.72	35.99	24035.84	2.38	0.60	0.77
$$n = 5000$$									
K-PIR (ls)	0.03	0.00	0.09	0	0.04	81.46	0.17	0.04	0.06
K-PIR (mle)	0.03	0.00	0.09	0	0.03	80.33	0.15	0.04	0.05
K-PFC1	0.03	0.00	0.09	0	0.04	79.23	0.17	0.04	0.06
K-PFC2	0.28	0.11	0.09	0	2.95	79.23	5.49	1.82	1.82
K-PFC3	0.42	0.25	0.09	0	7.16	79.23	5.49	1.82	1.82
(2D)${^2}$PCA	1.41	0.02	86.52	0.74	36.00	18615.04	2.16	0.53	0.70

$$E_1 = \Vert {\widehat{\varvec{\alpha }}} \otimes {\widehat{\varvec{\beta }}} - \varvec{\alpha }\otimes \varvec{\beta }\Vert / \Vert \varvec{\alpha }\otimes \varvec{\beta }\Vert $$, $$E_2 = \Vert {\widehat{\varvec{\varDelta }}} - \varvec{\varDelta }\Vert / \Vert \varvec{\varDelta }\Vert $$,

$$\Phi = \Vert {\widehat{\varvec{\varGamma }}}{\widehat{\varvec{\varGamma }}}^T - \varvec{\varGamma }\varvec{\varGamma }^T\Vert $$, $$\phi _i = \Vert {\widehat{\varvec{\varGamma }}}_i{\widehat{\varvec{\varGamma }}}_i^T - \varvec{\varGamma }_i\varvec{\varGamma }_i^T\Vert , i = 1, 2$$

**Table 2:** Continuous outcomes *Y* with $$ p = 10, T = 8, r = k = 6$$ and $${\text {rank}}(\varvec{\alpha }) = {\text {rank}}(\varvec{\beta }) < 6$$

Method	Mean $E_1$	SD $E_1$	Mean $E_2$	SD $E_2$	$V_1$	$V_2$	$\Phi$	$\phi _1$	$\phi _2$
$$n = 500, {\text {rank}}(\varvec{\alpha }) = {\text {rank}}(\varvec{\beta }) = 4$$									
K-PIR (ls)	0.17	0.01	0.32	0.01	0.48	105.40	0.59	0.18	0.23
K-PIR (mle)	0.13	0.02	0.29	0.01	0.28	91.32	0.48	0.15	0.18
K-PFC1	0.14	0.01	0.28	0.01	0.33	86.14	0.58	0.18	0.23
K-PFC2	0.50	0.11	0.28	0.01	3.42	86.14	3.47	1.36	1.37
K-PFC3	0.68	0.22	0.28	0.01	5.22	86.14	3.47	1.36	1.37
(2D)${^2}$PCA	NA	NA	69.17	1.51	NA	18182.77	2.91	1.00	1.12
$$n = 5000, {\text {rank}}(\varvec{\alpha }) = {\text {rank}}(\varvec{\beta }) = 4$$									
K-PIR (ls)	0.05	0.00	0.09	0	0.04	81.46	0.18	0.06	0.07
K-PIR (mle)	0.05	0.01	0.09	0	0.03	80.33	0.18	0.06	0.07
K-PFC1	0.04	0.00	0.09	0	0.03	79.86	0.18	0.06	0.07
K-PFC2	0.25	0.14	0.09	0	1.27	79.86	3.36	1.31	1.30
K-PFC3	0.37	0.28	0.09	0	3.04	79.86	3.36	1.31	1.30
(2D)${^2}$PCA	NA	NA	69.30	0.61	NA	14829.42	2.66	0.90	1.02
$$n = 500, {\text {rank}}(\varvec{\alpha }) = {\text {rank}}(\varvec{\beta }) = 2$$									
K-PIR (ls)	0.36	0.03	0.32	0.01	0.51	105.38	0.50	0.23	0.27
K-PIR (mle)	0.28	0.03	0.29	0.01	0.31	91.22	0.44	0.20	0.24
K-PFC1	0.19	0.02	0.29	0.01	0.15	90.31	0.47	0.22	0.25
K-PFC2	0.45	0.26	0.29	0.01	1.00	90.31	1.37	0.69	0.71
K-PFC3	0.50	0.30	0.29	0.01	1.19	90.31	1.37	0.69	0.71
(2D)${^2}$PCA	NA	NA	59.55	1.33	NA	15246.36	2.42	1.34	1.43
$$n = 5000, {\text {rank}}(\varvec{\alpha }) = {\text {rank}}(\varvec{\beta }) = 2$$									
K-PIR (ls)	0.10	0.01	0.09	0	0.04	81.46	0.15	0.07	0.08
K-PIR (mle)	0.10	0.01	0.09	0	0.03	80.33	0.16	0.07	0.08
K-PFC1	0.06	0.01	0.09	0	0.01	80.25	0.15	0.07	0.08
K-PFC2	0.20	0.21	0.09	0	0.33	80.25	1.22	0.61	0.61
K-PFC3	0.22	0.22	0.09	0	0.37	80.25	1.22	0.61	0.61
(2D)${^2}$PCA	NA	NA	59.67	0.54	NA	12740.16	2.36	1.29	1.37

$$E_1 = \Vert {\widehat{\varvec{\alpha }}} \otimes {\widehat{\varvec{\beta }}} - \varvec{\alpha }\otimes \varvec{\beta }\Vert / \Vert \varvec{\alpha }\otimes \varvec{\beta }\Vert $$, $$E_2 = \Vert {\widehat{\varvec{\varDelta }}} - \varvec{\varDelta }\Vert / \Vert \varvec{\varDelta }\Vert $$, $$\Phi = \Vert {\widehat{\varvec{\varGamma }}}{\widehat{\varvec{\varGamma }}}^T - \varvec{\varGamma }\varvec{\varGamma }^T\Vert $$, $$\phi _i = \Vert {\widehat{\varvec{\varGamma }}}_i{\widehat{\varvec{\varGamma }}}_i^T - \varvec{\varGamma }_i\varvec{\varGamma }_i^T\Vert , i = 1, 2$$

**Table 3:** Binary outcome *Y* with $p = 10, T = 5, r = k = 1$, ${\text {rank}}(\varvec{\alpha }) = {\text {rank}}(\varvec{\beta }) = 1$

Method	Mean $E_1$	SD $E_1$	Mean $E_2$	SD $E_2$	$V_1$	$V_2$	$\Phi$	$\phi _1$	$\phi _2$
$$n = 500, \rho _T = \rho _p = 0.3$$									
K-PIR (ls)	2.02	0.11	0.28	0.01	0.16	5.06	0.42	0.24	0.34
K-PIR (mle)	2.01	0.11	0.28	0.01	0.17	5.05	0.44	0.26	0.35
K-PFC1	2.02	0.11	0.28	0.01	0.16	5.04	0.42	0.24	0.34
K-PFC2	0.84	0.78	0.28	0.01	1.38	5.04	0.41	0.24	0.33
K-PFC3	0.84	0.78	0.28	0.01	1.38	5.04	0.41	0.24	0.33
LSIR	1.48	0.26	1.00	0.00	0.27	0.00	0.81	0.55	0.63
$$n = 1000, \rho _T = \rho _p = 0.3$$									
K-PIR (ls)	2.02	0.08	0.2	0.01	0.08	2.54	0.30	0.17	0.24
K-PIR (mle)	2.01	0.08	0.2	0.01	0.08	2.53	0.30	0.17	0.24
K-PFC1	2.02	0.08	0.2	0.01	0.08	2.53	0.30	0.17	0.24
K-PFC2	0.64	0.75	0.2	0.01	1.12	2.53	0.29	0.17	0.24
K-PFC3	0.64	0.75	0.2	0.01	1.12	2.53	0.29	0.17	0.24
LSIR	1.53	0.17	1.0	0.00	0.13	0.00	0.67	0.49	0.48
$$n = 2000, \rho _T = \rho _p = 0.3$$									
K-PIR (ls)	2.00	0.05	0.14	0	0.04	1.27	0.22	0.12	0.18
K-PIR (mle)	2.00	0.06	0.14	0	0.04	1.27	0.22	0.12	0.17
K-PFC1	2.00	0.05	0.14	0	0.04	1.27	0.22	0.12	0.18
K-PFC2	0.40	0.62	0.14	0	0.70	1.27	0.22	0.12	0.17
K-PFC3	0.40	0.62	0.14	0	0.70	1.27	0.22	0.12	0.17
LSIR	1.56	0.01	1.00	0	0.04	0.00	0.59	0.47	0.38

$$E_1 = \Vert {\widehat{\varvec{\alpha }}} \otimes {\widehat{\varvec{\beta }}} - \varvec{\alpha }\otimes \varvec{\beta }\Vert / \Vert \varvec{\alpha }\otimes \varvec{\beta }\Vert $$, $$E_2 = \Vert {\widehat{\varvec{\varDelta }}} - \varvec{\varDelta }\Vert / \Vert \varvec{\varDelta }\Vert $$,

$$\Phi = \Vert {\widehat{\varvec{\varGamma }}}{\widehat{\varvec{\varGamma }}}^T - \varvec{\varGamma }\varvec{\varGamma }^T\Vert $$, $$\phi _i = \Vert {\widehat{\varvec{\varGamma }}}_i{\widehat{\varvec{\varGamma }}}_i^T - \varvec{\varGamma }_i\varvec{\varGamma }_i^T\Vert , i = 1, 2$$

As a measure of variability, we calculated $V_1$, the trace of the empirical covariance matrix of $\text {vec}({\widehat{\varvec{\alpha }}}_i\otimes {\widehat{\varvec{\beta }}}_i)$, a $pTrk\times 1$ vector, for $i = 1, \ldots ,N=500$ repetitions for each simulation setting. Similarly, we computed the trace of the empirical covariance matrix of $\text {vec}({\widehat{\varvec{\varDelta }}})$, $V_2$, as a measure of variability of the estimates of the covariance matrix $\varvec{\varDelta }$.

The accuracy of the estimation is assessed by the Frobenius norm of the difference of the projections to the relative spans of the true and the estimated dimension reduction matrices.^4^ We report averages over 500 replicates of the following: $\Phi =\Vert \mathbf {P}_{{\widehat{{\varvec{\scriptstyle \varGamma }}}}}- \mathbf {P}_{{\varvec{\scriptstyle \varGamma }}}\Vert$, and $\phi _i = \Vert \mathbf {P}_{{\widehat{{\varvec{\scriptstyle \varGamma }}}}_i}-\mathbf {P}_{{\varvec{\scriptstyle \varGamma }}_i}\Vert , i= 1, 2$, where $\mathbf {P}_{\mathbf {A}}=\mathbf {A}(\mathbf {A}^T\mathbf {A})^{-1}\mathbf {A}^T$ is the orthogonal projection onto the span of a full-rank matrix $\mathbf {A}$.

### Variable selection

#### Data generation

To assess the performance of the variable selection method in Sect. 4.4, we generated continuous outcome data by first generating $y_i \sim N(0,1)$ for $i =1,\ldots ,n$, and then computed the *i*th row $\mathbf {f}_{y_i} = \mathbf {g}_{y_i}-{\bar{\mathbf {g}}}$ of the $n\times rk$ matrix ${\mathbb {F}}_y$, where $\mathbf {g}_{y}=(1,y,y^2)$. The $n \times pT$ matrix of error terms, ${\mathbb {E}}$, was generated from the multivariate normal $N_{npT}(\mathbf {0}, \varvec{\varDelta }\otimes \mathbf {I}_n)$, where $\varvec{\varDelta }$ was a positive definite matrix with ones on the diagonal. We then computed ${\mathbb {X}}= {\mathbb {F}}_{y} (\varvec{\alpha }\otimes \varvec{\beta })^T + \varvec{\varepsilon }$, where the $2 \times T$ matrix $\varvec{\alpha }$ had entries $\alpha _{11}=\alpha _{22}=1$ and all other entries $\alpha _{ij}, i=1, 2, j=1, \ldots , T$ were zero, and $\varvec{\beta }$ was a vector of length *p* with $\beta _1=1$ and $\beta _i=0, i=2, \ldots , p$ for $p=10$ and $T=5$.

We evaluated the influence of the sample size, *n*, and magnitude of noise, by multiplying the error term $\varvec{\varepsilon }$ in the linear model by a constant factor, called “Scale” in Table 4.

#### Performance criteria for variable selection

We computed how often markers (rows) and time points (columns) of $\mathbf {X}$ were correctly selected on average.

The following quantities are reported. *False positives (FPs):* An FP occurs when $\varvec{\alpha }_{ij}=0$, but its estimate ${\widehat{\varvec{\alpha }}}_{ij} \ne 0$. The FP rate for $\varvec{\alpha }$ is the percentage of times an FP occurs for $\varvec{\alpha }_{ij}$, and the overall FP rate (FPR) is the average of the FPRs across all zero coefficients of $\varvec{\alpha }$. *False negatives (FNs):* An FN occurs when $\varvec{\alpha }_{ij} \ne 0$, but its estimate ${\widehat{\varvec{\alpha }}}_{ij} =0$. The FN rate for $\varvec{\alpha }$ is the percentage of times an FN occurs for $\varvec{\alpha }_{ij}$, and the overall FN rate (FNR) is the average of the FN rates across all nonzero coefficients of $\varvec{\alpha }$. The *total error rate* is computed as the sum of the times a nonzero coefficient of $\varvec{\alpha }$ was estimated to be zero and the times a zero coefficient was estimated to be nonzero, divided by the total number of elements in $\varvec{\alpha }$.

The corresponding FPR, FNR and total error rate for $\varvec{\beta }$ are reported separately.

### Results for continuous outcome *Y*

We present results for $p=10$ and $T=8$ in Tables 1 and 2. Results for other values of *p* and *T* were qualitatively similar.

Table 1 shows summary performance statistics when $r=k=6$ and both $\varvec{\alpha }$ and $\varvec{\beta }$ are of full rank 6 for $n=500, 5000$. In this setting, the K-PIR (mle) estimates of $\varvec{\alpha }\otimes \varvec{\beta }$ had lower bias ($E_1$) and distance between subspaces ($\Phi , \phi _1$ and $\phi _2)$ than those for all other algorithms for $n=500$. K-PFC1 and K-PIR (ls) estimates of $\varvec{\alpha }\otimes \varvec{\beta }$ were similar with respect to all measures, but K-PIR (ls) estimates of $\varvec{\varDelta }$ had a larger bias and more variability than those of K-PFC1. K-PFC2 and K-PFC3 resulted in significantly larger bias and lower estimation accuracy measures for both sample sizes. (2D)$^2$PCA-based estimates of $\varvec{\alpha }\otimes \varvec{\beta }$ had much larger bias and variability than all other methods, but had the resulting estimates had smaller distance to the true subspace than K-PFC2, K-PFC3.

Table 2 shows results for $r=k=6$ for the non-full-rank case. While the general patterns were similar to the full-rank setting, all methods had poorer performance. For ${\text {rank}}(\varvec{\alpha })={\text {rank}}(\varvec{\beta })=4$ and $n=500$ K-PIR (mle) yielded the least biased estimates of $\varvec{\alpha }\otimes \varvec{\beta }$ and the smallest distances $\Phi , \phi _1$ and $\phi _2$. K-PFC1 was slightly better than K-PIR (ls) in terms of bias of $\varvec{\alpha }\otimes \varvec{\beta }$. For $n=5000$, K-PIR (ls), K-PIR (mle) and K-PFC1 estimates all had the same performance.

When ${\text {rank}}(\varvec{\alpha })={\text {rank}}(\varvec{\beta })=2$, however, K-PFC1-based estimates of $\varvec{\alpha }\otimes \varvec{\beta }$ had much lower bias, variability and distance to the true subspace and also better estimated $\varvec{\varDelta }$ than all other methods.

For all parameter settings and sample sizes, K-PFC2- and K-PFC3-based estimates were very similar and resulted in poorer estimation than the other three methods. (2D)$^2$PCA does not yield estimates for $\varvec{\alpha }$ and $\varvec{\beta }$ in the non-full-rank case. With respect to other measures, it behaved similarly to the full-rank case.

### Results for binary outcome *Y*

We present results for $p=10$ and $T=5$ in Table 3. Findings were qualitatively similar for other choices of *p* and *T*. The sample size *n* refers to the number of samples in each of the $Y=0$ and the $Y=1$ groups. Interestingly, in contrast to the results for continuous outcome, for all sample sizes estimates of $\varvec{\alpha }\otimes \varvec{\beta }$ and $\varvec{\varDelta }$ from K-PFC2 and K-PFC3 had the lowest bias and the smallest variance of all methods. The K-PFC2- and K-PFC3-based estimates also had slightly better performance in estimating the subspaces for smaller sample sizes, but for larger *n* all methods resulted in similar performance of the estimates. LSIR-based estimates [34] had larger bias and variance estimates compared to those from K-PFC2 and K-PFC3, but smaller compared to estimates from K-PIR (ls), K-PIR (mle) and K-PFC1 for all sample sizes. However, LSIR had worse performance than all other methods in estimating subspaces for all sample sizes.

**Table 4:** Sparse case: FNR = false negative rate, FPR = false positive rate. The nonzero entries of $\varvec{\alpha }$ and $\varvec{\beta }$ had values equal to one. “Scale” corresponds to a term that multiplied the standard deviation of the noise term in the data and reflects the noise-to-signal ratio

	$\varvec{\alpha }$			$\varvec{\beta }$		
Scale	Mean	Mean	Total	Mean	Mean	Total
	FNR	FPR	Error rate	FNR	FPR	Error rate
$$n = 100, (p, t, k, r) = (10, 5, 1, 2)$$						
1	0.000	0.000	0.000	0.000	0.002	0.002
2	0.008	0.000	0.003	0.000	0.036	0.033
3	0.063	0.030	0.043	0.014	0.069	0.064
4	0.204	0.105	0.145	0.116	0.104	0.105
5	0.295	0.141	0.202	0.188	0.134	0.139
$$n = 500, (p, t, k, r) = (10, 5, 1, 2)$$						
1	0.000	0.000	0.000	0	0.000	0.000
2	0.000	0.000	0.000	0	0.000	0.000
3	0.000	0.000	0.000	0	0.008	0.007
4	0.004	0.000	0.002	0	0.028	0.026
5	0.007	0.001	0.004	0	0.040	0.036

### Results for variable selection

In Table 4, we present results on the accuracy of our variable selection approach. For both $n=100, 500$ with $p=10$ and $T=5$, the false negative rate (FNR) was 0 for $\varvec{\alpha }$ and $\varvec{\beta }$ for low noise-to-signal ratio. For $n=100$ and at the highest signal-to-noise ratio we report, the FNR jumped to 29.5% for $\varvec{\alpha }$ and 18.8% for $\varvec{\beta }$, with lower false positive rates (FPR=14.1% for $\varvec{\alpha }$ and FPR=13.4% for $\varvec{\beta }$). The total error rates was 20.2% and 13.4% for $\varvec{\alpha }$ and $\varvec{\beta }$, respectively. For the more realistic setting of noise with 3 times the magnitude of the mean parameters, all error rates were less than 7% for both matrices.

When the sample size was increased to $n=500$, even when the noise standard deviation was 5 times larger than the magnitude of the mean parameters, all error rates for both matrices were below 5%, indicating excellent performance in variable selection.

## Serially measured pre-diagnostic levels of serum biomarkers and risk of brain cancer

To illustrate our methods, we used data from 128 individuals diagnosed with glioma, a type of brain cancer (cases, $Y=1$) and 111 healthy individuals (controls, $Y=0$) from a study that assessed the associations of fourteen serially measured biomarkers with glioma risk in individuals sampled from active component military personnel [2]. The markers were measured in serum obtained at three time points prior to diagnosis for cases, or selection for controls. The serum was typically what remains after routine, periodic HIV testing or required pre- and post-deployment samples. On average, samples were available every two years for a given person.

We analyzed the log-transformed values of 13 markers, including several interleukins (ILs), IL-12p40, IL-15, IL-16, IL-7, IL-10, monocyte chemoattractant protein (MCP1), thymus and activation regulated chemokine (TARC), placental growth factor (PLGF), vascular endothelial growth factor (VEGF), tumor necrosis factor alpha (TNFa), hepatocyte growth factor (HGF), interferon gamma (IFN$\gamma$) and transforming growth factor beta (TGFb1). One marker (IL8) that a had highly non-normal distribution, even after log transformation, was excluded from the original panel in order to allow comparison with K-MLE, resulting in $(p,T)= (13,3)$. We also compared all proposed methods with LSIR [34].

The discriminatory ability of the linear combinations from the various approaches to distinguish the two groups $Y=0$ and $Y=1$ was assessed by the area under the receiver operator characteristics curve, AUC [33, p. 67]. We used leave-one-out cross-validation to obtain an unbiased AUC estimate. That is, we removed person *i* from the data set, estimated the parameters of the respective model from the remaining samples and computed the projections of $\mathbf {X}_i$ onto the respective SDR subspace for person *i*. We repeated these steps by letting *i* range from 1 to the total sample size, to obtain unbiased predictions. For binary *Y*, all methods estimate at most a single direction in the central subspace; i.e., ${\mathcal {S}}_{\text {FMSDR}}$ is a vector. We thus used the projections onto the space spanned by the core matrices of the methods directly as a scalar diagnostic score in computing the AUC and its variance with the R package pROC [36].

Table 5 reports AUC values and their standard deviations. All of our proposed methods had the same discriminatory ability, with an AUC values of 0.66 for K-PIR, K-PFC1, K-PFC2 and K-PFC3 and for K-PIR (mle). LSIR, which assumes the Kronecker product structure for the first and the second moments of $\mathbf {X}$, had the highest AUC, AUC=0.69 highlighting the impact of further reducing complexity of estimating the central subspace, especially in settings of limited sample size.

**Table 5:** Mean AUC values and their standard deviations (St. Dev.) based on leave-one-out cross-validation for cytokine data ($p=13$, $T=3$) for 128 glioma cases and 111 control subjects

	AUC	St. Dev.
K-PIR (ls)	0.66	0.04
K-PIR (mle)	0.66	0.04
K-PFC1	0.66	0.04
K-PFC2	0.66	0.04
K-PFC3	0.66	0.04
LSIR	0.69	0.03

**Table 6:** Mean AUC values and their standard deviation based on tenfold cross-validation for the EEG imaging data (77 alcoholic and 45 control subjects)

	Method	AUC	St. Dev.
$T^{\star }=3,p^{\star }=4$	K-PIR (ls)	0.78	0.04
	K-PIR (mle)	0.75	0.05
	K-PFC1	0.78	0.04
	K-PFC2	0.78	0.04
	LSIR	0.85	0.04
	(2D)${^2}$PCR	0.83	0.04
$T^{\star }=15,p^{\star }=15$	K-PIR (ls)	0.78	0.04
	K-PIR (mle)	0.78	0.04
	K-PFC1	0.78	0.04
	K-PFC2	0.78	0.04
	LSIR	0.81	0.04
	(2D)${^2}$PCR	0.50	0.05
$T^{\star }=20,p^{\star }=30$	K-PIR (ls)	0.78	0.04
	K-PIR (mle)	0.77	0.04
	K-PFC1	0.78	0.04
	K-PFC2	0.78	0.04
	LSIR	0.83	0.04
	(2D)${^2}$PCR	0.53	0.05
$T=256,p=64$	FastPOI-C	$0.63^*$	0.22

$${}^* $$Mean AUC over the tenfold

## EEG Data

For the second example, we analyzed EEG data from a small study of 77 alcoholic and 45 control subjects (http://kdd.ics.uci.edu/databases/eeg/eeg.data.html). The data for each study subject consisted of a $64 \times 256$ matrix, with each column representing a time point and each row a channel. The measurements were obtained by exposing each individual to visual stimuli and measuring voltage values from 64 electrodes placed on the subjects’ scalps sampled at 256 time points (at 256 Hz for 1 second). Different stimulus conditions were used, and for each condition, 120 trials were measured.

**Fig. 2:**
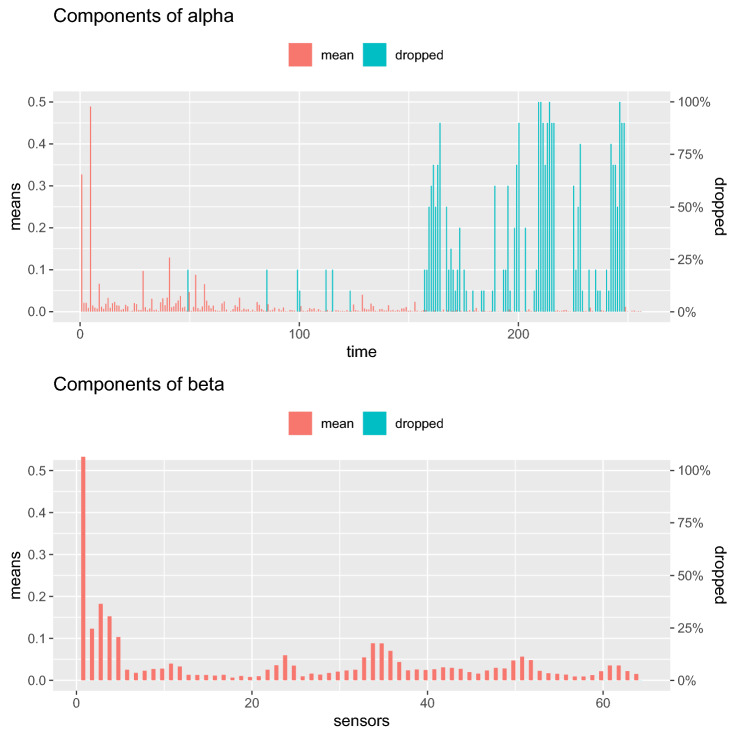
$\varvec{\alpha }, \varvec{\beta }$ components from tenfold EEG analysis

To facilitate comparison of our results with other published analyses, we used only a single stimulus condition (S1), and for each subject, we took the average of all the trials under that condition. That is, we used $(\mathbf {X}_i, Y_i), i=1,\ldots , 122$, where $\mathbf {X}_i$ is a $64 \times 256$ matrix, i.e., $p=64, T=256$, with each entry representing the mean voltage value of subject *i* at a combination of a time point and a channel, averaged over all trials under the S1 stimulus condition, and *Y* was a binary outcome variable with $Y = 1$ for an alcoholic and $Y = 0$ for a control subject. The $p T \times pT= 16384 \times 16384$ sample variance–covariance matrix of the predictors (${\widehat{\varvec{\varSigma }}}_{\mathbf {x}}$) is singular, since the sample size is 122.

We carried out two separate analyses. First, to bypass the issue of *large* *p* *small* *n*, we applied the same pre-screening procedure as in [29], which is a version of (2D)$^2$PCA [45], to reduce the order to $(p^{\star },T^{\star })=(30,20)$, (15, 15) and (4, 3). The pre-screened data were computed by replacing the matrix predictors with their (2D)$^2$PCs, setting $\mathbf {X}_i^{\star } = \mathbf {U}_{\beta }^T \mathbf {X}_i \mathbf {U}_{\alpha }: p^{\star } \times T^{\star }$, $i=1,\ldots , n$, as described in Sect. 5.

We used leave-one-out cross-validation to obtain unbiased estimates of the AUC. Results for $(p^{\star },T^{\star })= (4,3)$, (15,15), (30,20) are given in Table 6 for K-PFC1, K-PFC2, K-PIR (ls) and K-PIR (mle). K-PFC3 is identical to K-PFC2 in this example and thus not shown. These methods resulted in highly discriminating linear combinations, with AUC values of 0.78 for all choices of $p^{\star }$ and $T^{\star }$, except for K-PIR (mle) with AUC values of 0.75 and 0.77 for $(p^{\star },T^{\star })= (4,3)$, and (30,20), respectively.

(2D)$^2$PCR linear combinations had a highly variable performance, ranging from AUC of 0.83 for $(p^{\star },T^{\star })=(4,3)$ to 0.50 for $(p^{\star },T^{\star })=(30,20)$. AUC values did not decrease monotonically (unreported results), indicating lack of stability of the method. LSIR [34] linear combinations resulted in the best discriminatory performance and higher AUC values than all other methods for our choices of $(p^{\star }, T^{\star })$.

[29] analyzed these data with their method, *folded SIR*, also using $(p^{\star },T^{\star })= (15,15)$. In contrast to our algorithms, folded SIR uses starting values for $\varvec{\alpha }$ and $\varvec{\beta }$ that are random draws from two multivariate normal distributions, which results in different estimates every time the method is applied. We repeated the analysis using folded SIR several times and obtained consistently lower AUC values than with our methods, ranging from 0.61 to 0.70, which also reflects the numerical instability of the folded SIR estimation algorithm.

We also applied coordinate-wise sparse SDR without preprocessing the data, as described in Sect. 4.4, to simultaneously identify important variables and sufficient reductions. We report the average AUC values and corresponding standard deviations from tenfold cross-validation (due to the computational burden) fast POI-C [26] in the last row of Table 6. The average AUC value was 0.63, much lower than the AUCs from all other estimation methods.

Figure 2 plots the mean values of the estimated sparse $\varvec{\alpha }$ (top panel) and $\varvec{\beta }$ (bottom panel) components over the tenfold. The right *y*-axis shows the percent times the component was dropped. No components of $\varvec{\beta }$ were consistently dropped indicating that no specific sensor was found to be insignificant. In contrast, approximately 40% of the later time points were consistently dropped. That is, sparse SDR identifies the earlier time measurements to be more predictive of alcoholism status.

## Discussion

In this paper, we propose methods for regression and classification with matrix-valued predictors that yield consistent estimators, which are also asymptotically optimal when the predictors given the outcome have exponential family distributions. The least squares estimation algorithms are fast with guaranteed convergence. Our methods can incorporate simultaneous variable selection in estimating the sufficient dimension reduction, which further reduces complexity.

The dimensions $d_1$ and $d_2$ of ${\text {span}}(\varvec{\alpha })$ and ${\text {span}}(\varvec{\beta })$, respectively, are assumed to be known in our computations. Their estimation can be carried out, for example, via AIC and BIC [17]. Our methodology can be extended to regressions with multidimensional array-valued predictors.

The R code that implements the methods in this paper can be downloaded from https://git.art-ist.cc/daniel/tensor_predictors/releases.

## Appendix

*Van Loan and Pitsianis Matrix Approximation:* Van Loan and Pitsianis [40] proposed a singular value decomposition-based algorithm to efficiently find the optimal factor matrices $\mathbf {B}$ and $\mathbf {C}$ that minimize the Frobenius norm $\Vert \mathbf {A}-\mathbf {B}\otimes \mathbf {C}\Vert$, where $\mathbf {A}: p \times q$, $\mathbf {B}: p1 \times q1$, $\mathbf {C}: p_2 \times q_2$ with $p=p_1p_2$ and $q=q_1q_2$. They write $\mathbf {A}$ as a $p_1p_2 \times q_1q_2$ block matrix,

$$\begin{aligned} \mathbf {A}= \begin{pmatrix} \mathbf {A}_{11} &{} \mathbf {A}_{12} &{} \cdots &{} \mathbf {A}_{1,q_1} \\ \mathbf {A}_{21} &{} \mathbf {A}_{22} &{} \cdots &{} \mathbf {A}_{2,q_1} \\ \vdots &{} \vdots &{} \ddots &{} \vdots \\ \mathbf {A}_{p_1,1} &{} \mathbf {A}_{p_1,2} &{} \cdots &{} \mathbf {A}_{p_1,q_1} \end{pmatrix} \end{aligned}$$

where $\mathbf {A}_{ij}: p_2 \times q_2$, and show that the Kronecker product approximation for two factor matrices is equivalent to finding a nearest rank 1 matrix to ${\mathcal {R}}(\mathbf {A})$,

$$\begin{aligned} \Vert \mathbf {A}-\mathbf {B}\otimes \mathbf {C}\Vert = \Vert {\mathcal {R}}(\mathbf {A}) -\text {vec}(\mathbf {B})\text {vec}(\mathbf {C})^T\Vert \end{aligned}$$

with

$$\begin{aligned} {\mathcal {R}}(\mathbf {A}) = \begin{pmatrix} \mathbf {A}_1 \\ \mathbf {A}_2 \\ \vdots \\ \mathbf {A}_{q_1} \end{pmatrix}, \; \mathbf {A}_j=\begin{pmatrix} \text {vec}(\mathbf {A}_{1,j})^T \\ \text {vec}(\mathbf {A}_{2,j})^T \\ \vdots \\ \text {vec}(\mathbf {A}_{p_1,j})^T \end{pmatrix}\end{aligned}$$

for $j=1,\ldots , q_1$. This problem can be solved by singular value decomposition [22], as follows. If the SVD of ${\mathcal {R}}$ is $\mathbf {U}^T{\mathcal {R}}\mathbf {V}=\varvec{\varLambda }={\text {diag}}(\lambda _1,\ldots ,\lambda _{\min (p,q)})$, the optimal $\mathbf {B}$ equals $\sqrt{\lambda _1}\mathbf {U}_1$ and the optimal $\mathbf {C}$, $\sqrt{\lambda _1}\mathbf {V}_1$, where $\mathbf {U}_1, \mathbf {V}_1$ are the first columns of $\mathbf {U}$ and $\mathbf {V}$, respectively.

### Proof of Theorem 1

Suppose the true parameter matrix has the form $\mathbf {B}^T = \varvec{\alpha }\otimes \varvec{\beta }$, where $\varvec{\alpha }\in {{\mathbb {R}}}^{T \times r}$, and $\varvec{\beta }\in {\mathbb {R}}^{p\times k}$. Thus,

33 $$\begin{aligned} \mathbf {B}^T&= \left( \begin{array}{ccc} \alpha _{11} \varvec{\beta }&{} \ldots &{} \alpha _{1r} \varvec{\beta }\\ \vdots &{} \ddots &{} \vdots \\ \alpha _{p1} \varvec{\beta }&{} \ldots &{} \alpha _{pr} \varvec{\beta }\\ \end{array}\right) = \left( \begin{array}{ccc} \mathbf {B}_{11} &{} \ldots &{} \mathbf {B}_{1r} \\ \vdots &{} \ddots &{} \vdots \\ \mathbf {B}_{p1} &{} \ldots &{} \mathbf {B}_{pr} \\ \end{array}\right) \end{aligned}$$

where $\mathbf {B}_{ij}= \alpha _{ij} \varvec{\beta }: T \times k$. In this proof, we assume the estimates ${\widehat{\varvec{\alpha }}}$ and ${\widehat{\varvec{\beta }}}$ are computed using the algorithm in Section 4 of [40], which is an alternating least squares algorithm for the calculation of the largest singular value of ${\mathcal {R}}(\mathbf {B}^T)$, as required in the Van Loan and Pitsianis Kronecker product matrix approximation [40]. That is, for fixed $\varvec{\beta }$,

$$\begin{aligned}{{\hat{\alpha }}}_{ij} = \frac{\text {tr}({{\widehat{\mathbf {B}}}}_{ij}^T \varvec{\beta })}{\text {tr}(\varvec{\beta }^T \varvec{\beta })}\end{aligned}$$

where ${{\widehat{\mathbf {B}}}}_{ij}$ is the lse of the corresponding true $\mathbf {B}_{ij}$ in (33). The approximation algorithm for ${\widehat{\varvec{\alpha }}}$ and ${\widehat{\varvec{\beta }}}$ is an *alternating least squares algorithm* and enjoys both global and local convergence [15]. Since the unconstrained least squares estimate ${\widehat{\mathbf {B}}}$ is consistent for $\mathbf {B}$, we obtain that ${{\widehat{\mathbf {B}}}}_{ij}$ is consistent for $\mathbf {B}_{ij}$, for all *i*, *j*, and

$$\begin{aligned}{{\hat{\alpha }}}_{ij} \rightarrow \frac{\text {tr}(\mathbf {B}_{ij}^T \varvec{\beta })}{\text {tr}(\varvec{\beta }^T \varvec{\beta })}= \frac{\text {tr}(\alpha _{ij} \varvec{\beta }^T \varvec{\beta })}{\text {tr}(\varvec{\beta }^T \varvec{\beta })}=\alpha _{ij} \frac{\text {tr}( \varvec{\beta }^T \varvec{\beta })}{\text {tr}(\varvec{\beta }^T \varvec{\beta })}=\alpha _{ij},\end{aligned}$$

and similarly ${{\hat{\beta }}}_{ij} \overset{p}{\rightarrow }\beta _{ij}$. Therefore, ${\widehat{\varvec{\alpha }}} \otimes {\widehat{\varvec{\beta }}} \overset{p}{\rightarrow }\varvec{\alpha }\otimes \varvec{\beta }$.

The unconstrained least squares estimate ${\widehat{\mathbf {B}}}$ is also asymptotically normal [3]. Therefore, each of its elements and any of its block matrices are asymptotically normal. Alternating least squares is a special case of Iteratively Reweighted Least Squares (IRLS), which yields MLEs for the normal distribution, as well as for all members of the exponential family because they are equivalent to Fisher’s scoring method [16, 23]. Thus, ${\widehat{\varvec{\alpha }}}$ and ${\widehat{\varvec{\beta }}}$ are also asymptotically normal. $\square$

*MLE Derivation:* We derive formulas (28), (29) and (7) for the MLEs of ${\mathcal {S}}_{{\varvec{\scriptstyle \varGamma }}_1 \otimes {\varvec{\scriptstyle \varGamma }}_2}$, $\varvec{\gamma }_1 \otimes \varvec{\gamma }_2$ and $\varvec{\varDelta }$, respectively.

Write $(\varvec{\varGamma }_1 \otimes \varvec{\varGamma }_2) (\varvec{\gamma }_1 \otimes \varvec{\gamma }_2)=\varvec{\varGamma }\varvec{\gamma }=\mathbf {B}^T$. Then, the full log-likelihood in (23) is

34 $$\begin{aligned} \ell _d(\varvec{\mu },S_{\varvec{\varGamma }},\varvec{\gamma },\varvec{\varDelta })&= -\frac{npT}{2}\log (2\pi )-(n/2)\log |\varvec{\varDelta }| \nonumber \\&\quad -\frac{1}{2}\sum \limits _{y}\left( \text {vec}(\mathbf {X}_y)-\text {vec}(\varvec{\mu }) -\varvec{\varGamma }\varvec{\gamma }{\widetilde{\mathbf {f}}}_y\right) ^T\nonumber \\&\qquad \varvec{\varDelta }^{-1}\left( \text {vec}(\mathbf {X}_y)-\text {vec}(\varvec{\mu }) -\varvec{\varGamma }\varvec{\gamma }{\widetilde{\mathbf {f}}}_y\right) \end{aligned}$$

Fixing $\varvec{\varDelta }$ and setting ${\widehat{\mathbf {B}}}=({\mathbb {F}}^T{\mathbb {F}})^{-1}{\mathbb {F}}^T {\mathbb {X}}$, [12] showed that the estimators ${\widehat{\varvec{\mu }}}={\bar{\mathbf {X}}}$, ${\widehat{{\mathcal {S}}}}_{{\varvec{\scriptstyle \varGamma }}} = \varvec{\varDelta }{\mathcal {S}}_d(\varvec{\varDelta },{\widehat{\varvec{\varDelta }}}_{\text {fit}})$, and ${\widehat{\varvec{\gamma }}} = ({\widehat{\varvec{\varGamma }}}^T\varvec{\varDelta }^{-1}{\widehat{\varvec{\varGamma }}})^{-1}{\widehat{\varvec{\varGamma }}}^{-T}\varvec{\varDelta }^{-1}{\widehat{\mathbf {B}}}^T$ are the MLEs of the corresponding parameters, where ${\widehat{\varvec{\varGamma }}}$ is any orthonormal basis for ${\widehat{{\mathcal {S}}}}_{{\varvec{\scriptstyle \varGamma }}}$. Here, ${\mathcal {S}}_d(\mathbf {A},\mathbf {B})$ denotes the span of $\mathbf {A}^{-1/2}$ times the first *d* eigenvectors of $\mathbf {A}^{-1/2}\mathbf {B}\mathbf {A}^{-1/2}$ for symmetric matrices $\mathbf {A}$ and $\mathbf {B}$.

Once ${\widehat{\varvec{\varGamma }}}$ is obtained, we can apply VLP to obtain ${\widehat{\varvec{\varGamma }}}={\widehat{\varvec{\varGamma }}}_1 \otimes {\widehat{\varvec{\varGamma }}}_2$, so that ${\widehat{\varvec{\varGamma }}}_1$ and ${\widehat{\varvec{\varGamma }}}_2$ are also orthogonal. Similarly for ${\widehat{\varvec{\gamma }}}$. We show next that the Kronecker product form of $\varvec{\varGamma }$ and $\varvec{\gamma }$ does not affect the MLE of $\varvec{\varDelta }$ in our setting.

Let ${\mathbb {S}}_q^+$ denote the set of $q \times q$ positive definite matrices. Substituting ${\widehat{\varvec{\mu }}},{\widehat{\varvec{\gamma }}}$ and ${\widehat{\varvec{\varGamma }}}$ in (34), the next step is to maximize

35 $$\begin{aligned} \ell _d(\varvec{\varDelta })&= -\frac{np}{2}\log (2\pi )-\frac{n}{2}\log |\varvec{\varDelta }| -\frac{n}{2}\text {tr}(\varvec{\varDelta }^{-1}{\widehat{\varvec{\varDelta }}}_{\text {res}}) \nonumber \\&\quad -\frac{n}{2}\sum \limits _{i=d+1}^{p}\lambda _i(\varvec{\varDelta }^{-1}{\widehat{\varvec{\varDelta }}}_{\text {fit}}) \end{aligned}$$

Following the derivation of the MLE of $\varvec{\varDelta }$ in [12], let $\mathbf {U}= {\widehat{\varvec{\varDelta }}}_{\text {res}}^{1/2}\varvec{\varDelta }^{-1}{\widehat{\varvec{\varDelta }}}_{\text {res}}^{1/2}$. Then $\text {tr}(\varvec{\varDelta }^{-1}{\widehat{\varvec{\varDelta }}}_{\text {res}}) = \text {tr}(\mathbf {U})$, and

$$\begin{aligned}\lambda _i(\varvec{\varDelta }^{-1}{\widehat{\varvec{\varDelta }}}_{\text {fit}}) = \lambda _i(\mathbf {U}{\widehat{\varvec{\varDelta }}}_{\text {res}}^{-1/2}{\widehat{\varvec{\varDelta }}}_{\text {fit}}{\widehat{\varvec{\varDelta }}}_{\text {res}}^{-1/2}), \end{aligned}$$

where $\lambda _i(\cdot )$ denotes the *i*th-order eigenvalue of the argument matrix, since $\text {tr}(\mathbf {A}\mathbf {B})=\text {tr}(\mathbf {B}\mathbf {A})$. Since these two matrices are similar and

$$\begin{aligned} |{\widehat{\varvec{\varDelta }}}_{\text {res}}^{1/2}| |\varvec{\varDelta }^{-1}| |{\widehat{\varvec{\varDelta }}}_{\text {res}}^{1/2}|&= |\mathbf {U}| = |{\widehat{\varvec{\varDelta }}}_{\text {res}}| |\varvec{\varDelta }^{-1}| = |{\widehat{\varvec{\varDelta }}}_{\text {res}}|\frac{1}{|\varvec{\varDelta }|}, \end{aligned}$$

maximizing (35) is equivalent to maximizing

36 $$\begin{aligned} f(\mathbf {U})&= \log |\mathbf {U}|-\text {tr}(\mathbf {U})-\sum \limits _{i=d+1}^p\lambda _i(\mathbf {U}{\widehat{\varvec{\varDelta }}}_{\text {res}}^{-1/2}{\widehat{\varvec{\varDelta }}}_{\text {fit}}{\widehat{\varvec{\varDelta }}}_{\text {res}}^{-1/2}) \end{aligned}$$

Let $\tau = min(rk,pT)$, where *pT* is the order of $\text {vec}(\mathbf {X})$ and *rk* is that of ${\widetilde{\mathbf {f}}}_y$, and consider the spectral value decomposition of ${\widehat{\varvec{\varDelta }}}_{\text {res}}^{-1/2}{\widehat{\varvec{\varDelta }}}_{\text {fit}}{\widehat{\varvec{\varDelta }}}_{\text {res}}^{-1/2} = {\widehat{\mathbf {V}}}{\widehat{\varvec{\varLambda }}}_{\tau }{\widehat{\mathbf {V}}}^T$, where ${\widehat{\mathbf {V}}}\in {\mathbb {R}}^{pT \times pT}$ is an orthogonal matrix and the diagonal ${\widehat{\varvec{\varLambda }}}_{\tau } = {\text {diag}}({\hat{\lambda }}_1,\ldots ,{\hat{\lambda }}_{\tau },0,\ldots ,0)$ with $\lambda _1\ge \lambda _2\ge \ldots \ge \lambda _{\tau }>0$. Let $\mathbf {H}= {\widehat{\mathbf {V}}}^T\mathbf {U}{\widehat{\mathbf {V}}}$. Then, $\mathbf {H}\in {\mathbb {S}}_{pT}^+$ and is similar to $\mathbf {U}$, so (36) yields

37 $$\begin{aligned} f(\mathbf {H}) = \log |\mathbf {H}|-\text {tr}|\mathbf {H}| - \sum \limits _{i=d+1}^{\tau }\lambda _i(\mathbf {H}{\widehat{\varvec{\varLambda }}}_\tau ) \end{aligned}$$

$$\begin{aligned} \sum \limits _{i=d+1}^{pT}\lambda _i(\mathbf {U}{\widehat{\varvec{\varDelta }}}_{\text {res}}^{-1/2}{\widehat{\varvec{\varDelta }}}_{\text {fit}}{\widehat{\varvec{\varDelta }}}_{\text {res}}^{-1/2}) = \sum \limits _{i=d+1}^{\tau }\lambda _i(\mathbf {H}{\widehat{\varvec{\varLambda }}}_\tau ) \end{aligned}$$

because

$$\begin{aligned} \sum \limits _{i=d+1}^{\tau }\lambda _i(\mathbf {H}{\widehat{\varvec{\varLambda }}}_\tau )&= \sum \limits _{i=d+1}^{pT}\lambda _i(\mathbf {H}{\widehat{\varvec{\varLambda }}}_\tau ) = \sum \limits _{i=d+1}^{pT}\lambda _i({\widehat{\mathbf {V}}}^T\mathbf {U}{\widehat{\mathbf {V}}}{\widehat{\varvec{\varLambda }}}_\tau ) \\&= \sum \limits _{i=d+1}^{pT}\lambda _i(({\widehat{\varvec{\varLambda }}}_\tau {\widehat{\mathbf {V}}}^T)(\mathbf {U}{\widehat{\mathbf {V}}}))\\&= \sum \limits _{i=d+1}^{pT}\lambda _i((\mathbf {U}{\widehat{\mathbf {V}}})({\widehat{\varvec{\varLambda }}}_\tau {\widehat{\mathbf {V}}}^T)) \\&= \sum \limits _{i=d+1}^{pT}\lambda _i(\mathbf {U}{\widehat{\varvec{\varDelta }}}_{\text {res}}^{-1/2}{\widehat{\varvec{\varDelta }}}_{\text {fit}}{\widehat{\varvec{\varDelta }}}_{\text {res}}^{-1/2}) \end{aligned}$$

We partition the positive definite matrix $\mathbf {H}$ as

38 $$\begin{aligned} \mathbf {H}= \begin{pmatrix}\mathbf {H}_{11}&{}\mathbf {H}_{12}\\ \mathbf {H}_{12}^T&{}\mathbf {H}_{22}\end{pmatrix} \end{aligned}$$

with $\mathbf {H}_{11}\in {\mathbb {S}}_{\tau }^+$,$\mathbf {H}_{22}\in {\mathbb {S}}_{pT-\tau }^+$ and consider the one to one and onto transformation [18, Prop. 5.8],

39 $$\begin{aligned} \mathbf {H}\longrightarrow \begin{pmatrix}\mathbf {H}_{11}&{}\mathbf {H}_{12}^T\\ 0&{}\mathbf {H}_{22}-\mathbf {H}_{12}^T\mathbf {H}_{11}^{-1}\mathbf {H}_{12}\end{pmatrix}. \end{aligned}$$

Let $\mathbf {V}_{11} = \mathbf {H}_{11}$, $\mathbf {V}_{22} = \mathbf {H}_{22}-\mathbf {H}_{12}^T\mathbf {H}_{11}^{-1}\mathbf {H}_{12}$ and $\mathbf {V}_{12} = \mathbf {H}_{11}^{-11}\mathbf {H}_{12}$. By (39), $|\mathbf {H}| = |\mathbf {V}_{11}| |\mathbf {V}_{22}|$ and

$$\begin{aligned} \text {tr}(\mathbf {H})&= \text {tr}(\mathbf {H}_{11}) +\text {tr}(\mathbf {H}_{22}) \\&=\text {tr}(\mathbf {H}_{11})+ \text {tr}(\mathbf {H}_{22}-\mathbf {H}_{12}^T\mathbf {H}_{11}^{-1}\mathbf {H}_{12}) \\&\qquad + \text {tr}(\mathbf {H}_{12}^T\mathbf {H}_{11}^{-1}\mathbf {H}_{12})\\&= \text {tr}(\mathbf {V}_{11})+\text {tr}(\mathbf {V}_{22})+\text {tr}(\mathbf {V}_{12}^T\mathbf {V}_{11}\mathbf {V}_{12}) \end{aligned}$$

Since the nonzero eigenvalues of $\mathbf {H}{\widehat{\varvec{\varLambda }}}_{\tau }$ are the same as those of $\mathbf {H}_{11}{\tilde{\varvec{\varLambda }}}_{\tau }$, where ${\tilde{\varvec{\varLambda }}}_{\tau } = {\text {diag}}({\widehat{\lambda }}_1,\ldots ,{\widehat{\lambda }}_{\tau })$, (37) can be written as

40 $$\begin{aligned} \log |\mathbf {V}_{11}| |\mathbf {V}_{22}|-\text {tr}(\mathbf {V}_{11})-\text {tr}(\mathbf {V}_{22}) \nonumber \\ -\text {tr}(\mathbf {V}_{12}^T\mathbf {V}_{11}\mathbf {V}_{12}) -\sum \limits _{i=d+1}^{\tau }\lambda _i(\mathbf {V}_{11}{\tilde{\varvec{\varLambda }}}_{\tau }) \end{aligned}$$

Only the term $\text {tr}(\mathbf {V}_{12}^T\mathbf {V}_{11}\mathbf {V}_{12})$ in (40) depends on $\mathbf {V}_{12}$. Since $\mathbf {V}_{11}=\mathbf {H}_{11}$ is positive definite, $\mathbf {V}_{12}^T\mathbf {V}_{11}\mathbf {V}_{12}$ is positive semi-definite. Thus, the maximum occurs when $\mathbf {V}_{12}=\mathbf{0 }$. This implies that $\mathbf {H}_{12}=\mathbf{0 }$, $\mathbf {H}_{11} = \mathbf {V}_{11}$ and $\mathbf {H}_{22} = \mathbf {V}_{22}$. so (40), which is a function of $\mathbf {V}_{11},\mathbf {V}_{12}$ and $\mathbf {V}_{22}$, can be written as

41 $$\begin{aligned} f(\mathbf {H}_{11},\mathbf {H}_{22})&= \log |\mathbf {H}_{11}|+\log |\mathbf {H}_{22}|-\text {tr}(\mathbf {H}_{11}) \nonumber \\&\quad -\text {tr}(\mathbf {H}_{22})-\sum \limits _{i=d+1}^{\tau }\lambda _i(\mathbf {H}_{11}{\tilde{\varvec{\varLambda }}}_\tau ) \end{aligned}$$

$\mathbf {H}_{22} \in {\mathbb {S}}_{pT-\tau }^+$, $\mathbf {H}_{22}$ is similar to ${\text {diag}}(h_1,h_2,\ldots ,h_{pT-\tau })$ and

42 $$\begin{aligned}&\log |\mathbf {H}_{22}|-\text {tr}(\mathbf {H}_{22}) \nonumber \\&\quad = \log (h_1h_2...h_{pT-\tau })-(h_1+h_2+h_{pT-\tau }) \nonumber \\&\quad = \log (h_1)-h_1+\ldots +\log (h_{pT-\tau })-h_{pT-\tau } \end{aligned}$$

The maximum of $g(x) = \log x-x$ occurs at $x = 1$. Thus, (42) reaches its minimum for $h_i = 1$, $i=1,2,...,pT-\tau$, and $\mathbf {H}_{22}$ is an identity matrix when (42) is maximized. Next, for (41), we need to maximize

43 $$\begin{aligned} f(\mathbf {H}_{11})&= \log |\mathbf {H}_{11}|-\text {tr}(\mathbf {H}_{11})-\sum \limits _{i=d+1}^{\tau }\lambda _i(\mathbf {H}_{11}{\tilde{\varvec{\varLambda }}}_\tau ) \end{aligned}$$

Let $\mathbf {Z}= {\tilde{\varvec{\varLambda }}}_\tau ^{1/2}\mathbf {H}_{11}{\tilde{\varvec{\varLambda }}}_\tau ^{1/2}$. Following similar reasoning as from (35) to (36), maximizing (43) is equivalent to maximizing

44 $$\begin{aligned} f(\mathbf {Z})&= \log |\mathbf {Z}| -\text {tr}(\mathbf {Z}{\tilde{\varvec{\varLambda }}}_\tau ^{-1})-\sum \limits _{i=d+1}^{\tau }\lambda _i(\mathbf {Z}) \end{aligned}$$

Since $\mathbf {Z}\in {\mathbb {S}}_{\tau }^+$, there exists $\varvec{\Psi }= {\text {diag}}(\psi _1,\ldots ,\psi _{\tau })$ with $\psi _i>0$ and $\psi _1\ge \psi _2\ge \ldots \ge \psi _{\tau }$, and an orthogonal matrix $\mathbf {W}$ such that $\mathbf {Z}=\mathbf {W}^T\varvec{\Psi }\mathbf {W}$. We can rewrite (44) as a function of $\mathbf {W}$ and $\varvec{\Psi }$,

45 $$\begin{aligned} f(\varvec{\Psi },\mathbf {W})&= \log |\varvec{\Psi }|-\text {tr}(\mathbf {W}^T\varvec{\Psi }\mathbf {W}{\tilde{\varvec{\varLambda }}}_\tau ^{-1})-\sum \limits _{i=d+1}^{\tau }\psi _i \nonumber \\&= \log |\varvec{\Psi }|-\text {tr}(\varvec{\Psi }\mathbf {W}{\tilde{\varvec{\varLambda }}}_\tau ^{-1}\mathbf {W}^T)-\sum \limits _{i=d+1}^{\tau }\psi _i \end{aligned}$$

By [1, Thm. A.4.7] , $\min _{\mathbf {W}}\text {tr}(\varvec{\Psi }\mathbf {W}{\tilde{\varvec{\varLambda }}}_\tau ^{-1}\mathbf {W}^T) = \sum \limits _{i=1}^\tau \psi _i{\widehat{\lambda }}_i^{-1}$. If the diagonal elements of $\varvec{\Psi }$ and ${\tilde{\varvec{\varLambda }}}_\tau$ are distinct, the minimum occur when $\mathbf {W}=\mathbf {I}_{\tau }$. We can then rewrite (45) as a function of $\psi _i$, $i=1,2,\ldots ,\tau$, all greater than zero,

46 $$\begin{aligned} f(\psi _1,\ldots ,\psi _{\tau }) = \sum \limits _{i=1}^{\tau } \log \psi _i-\sum \limits _{i=1}^{\tau } \psi _i\widehat{\lambda _i}^{-1}-\sum \limits _{i=d+1}^{\tau }\psi _i \end{aligned}$$

The function $\log x-ax$ reaches its maximum when $x= 1/a$, for $a>0$. Therefore, (46) reaches its maximum when $\psi _i ={\widehat{\lambda }}_i$ for $i=1,2,\ldots ,d$ and $\psi _i = {\widehat{\lambda }}_i/(1+{\widehat{\lambda }}_i)$ for $i=d+1,\ldots ,\tau$. Since ${\widehat{\lambda }}_i$ are positive and in descending order, $\psi _i$ are positive, in descending order and distinct. Collecting all previous results, we obtain that the value of $\varvec{\varDelta }$ that maximizes (35) is

$$\begin{aligned} {\widehat{\varvec{\varDelta }}}_{\text {MLE}}&= {\widehat{\varvec{\varDelta }}}_{\text {res}}^{1/2}{\widehat{\mathbf {U}}}^{-1}{\widehat{\varvec{\varDelta }}}_{\text {res}}^{1/2} = {\widehat{\varvec{\varDelta }}}_{\text {res}}^{1/2}{\widehat{\mathbf {V}}}{\widehat{\mathbf {H}}}^{-1}{\widehat{\mathbf {V}}}^T{\widehat{\varvec{\varDelta }}}_{\text {res}}^{1/2}, \end{aligned}$$

where

47 $$\begin{aligned} \mathbf {H}= \begin{pmatrix}\mathbf {H}_{11}&{}\mathbf {H}_{12}\\ \mathbf {H}_{12}^T&{}\mathbf {H}_{22}\end{pmatrix} = \begin{pmatrix} {\tilde{\varvec{\varLambda }}}_\tau ^{1/2}{\widehat{\mathbf {Z}}}^{-1}{\tilde{\varvec{\varLambda }}}_\tau ^{1/2}&{}\mathbf{0 }_{\tau \times (p-\tau )}\\ \mathbf{0 }_{\tau \times (pT-\tau )}&{}\mathbf{I }_{(pT-\tau )\times (pT-\tau )} \end{pmatrix}, \end{aligned}$$

and $${\tilde{\varvec{\varLambda }}}_\tau ^{1/2}{\widehat{\mathbf {Z}}}^{-1}{\tilde{\varvec{\varLambda }}}_{\tau }^{1/2} = {\text {diag}}(\mathbf {I}_d,{\hat{\lambda }}_{d+1}+1,\ldots ,{\hat{\lambda }}_{\tau }+1)$$. $$\square $$

## Footnotes

1 $\varvec{\eta }^T\mathbf {X}=\varvec{\eta }^T\mathbf {O}^T \mathbf {O}\mathbf {X}$, for any orthogonal matrix $\mathbf {O}$.

2 From *pTkr* to $pk+Tr$.

3 The norm $\Vert \,.\,\Vert$ denotes the Frobenius norm, $\Vert \mathbf {A}\Vert = \left( \sum _{i,j} a_{i j}^2 \right) ^{1/2}$, for a matrix $\mathbf {A}=(a_{i j})$.

4 This is the optimal measure of distance between subspaces [43].
